# Interaction specificity and coexpression of rice NPR1 homologs 1 and 3 (NH1 and NH3), TGA transcription factors and Negative Regulator of Resistance (NRR) proteins

**DOI:** 10.1186/1471-2164-15-461

**Published:** 2014-06-11

**Authors:** Mawsheng Chern, Wei Bai, Deling Ruan, Taeyun Oh, Xuewei Chen, Pamela C Ronald

**Affiliations:** Department of Plant Pathology and the Genome Center, University of California, Davis, CA 95616 USA; College of Life Sciences, Inner Mongolia Agricultural University, Huhhot, 010018 China; Rice Institute, Sichuan Agricultural University at Chengdu, 211 Huimin Road, Liucheng, Wenjiang, Chengdu, Sichuan 611130 China

**Keywords:** SAR, Salicylic acid, NPR1, NH1, TGA, Protein interaction, Yeast two-hybrid, Split YFP, Co-expression, Innate immunity, Bridged split YFP, RH

## Abstract

**Background:**

The *nonexpressor of pathogenesis-related genes 1*, *NPR1* (also known as *NIM1* and *SAI1*), is a key regulator of SA-mediated systemic acquired resistance (SAR) in Arabidopsis. In rice, the NPR1 homolog 1 (NH1) interacts with TGA transcriptional regulators and the Negative Regulator of Resistance (NRR) protein to modulate the SAR response. Though five NPR1 homologs (NHs) have been identified in rice, only NH1 and NH3 enhance immunity when overexpressed. To understand why NH1 and NH3, but not NH2, NH4, or NH5, contribute to the rice immune response, we screened TGA transcription factors and NRR-like proteins for interactions specific to NH1 and NH3. We also examined their co-expression patterns using publicly available microarray data.

**Results:**

We tested five NHs, four NRR homologs (RHs), and 13 rice TGA proteins for pair-wise protein interactions using yeast two-hybrid (Y2H) and split YFP assays. A survey of 331 inter-family interactions revealed a broad, complex protein interaction network. To investigate preferred interaction partners when all three families of proteins were present, we performed a bridged split YFP assay employing YFPN-fused TGA, YFPC-fused RH, and NH proteins without YFP fusions. We found 64 tertiary interactions mediated by NH family members among the 120 sets we examined. In the yeast two-hybrid assay, each NH protein was capable of interacting with most TGA and RH proteins. In the split YFP assay, NH1 was the most prevalent interactor of TGA and RH proteins, NH3 ranked the second, and NH4 ranked the third. Based on their interaction with TGA proteins, NH proteins can be divided into two subfamilies: NH1, NH2, and NH3 in one family and NH4 and NH5 in the other.

In addition to evidence of overlap in interaction partners, co-expression analyses of microarray data suggest a correlation between NH1 and NH3 expression patterns, supporting their common role in rice immunity. However, NH3 is very tightly co-expressed with RH1 and RH2, while NH1 is strongly, inversely co-expressed with RH proteins, representing a difference between NH1 and NH3 expression patterns.

**Conclusions:**

Our genome-wide surveys reveal that each rice NH protein can partner with many rice TGA and RH proteins and that each NH protein prefers specific interaction partners. NH1 and NH3 are capable of interacting strongly with most rice TGA and RH proteins, whereas NH2, NH4, and NH5 have weaker, limited interaction with TGA and RH proteins in rice cells. We have identified rTGA2.1, rTGA2.2, rTGA2.3, rLG2, TGAL2 and TGAL4 proteins as the preferred partners of NH1 and NH3, but not NH2, NH4, or NH5. These TGA proteins may play an important role in NH1- and NH3-mediated immune responses. In contrast, NH4 and NH5 preferentially interact with TGAL5, TGAL7, TGAL8 and TGAL9, which are predicted to be involved in plant development.

**Electronic supplementary material:**

The online version of this article (doi:10.1186/1471-2164-15-461) contains supplementary material, which is available to authorized users.

## Background

Plants survive pathogen attack by employing various defense strategies, including strengthening their cell walls, accumulating phytoalexins, synthesizing salicylic acid (SA), and inducing pathogenesis-related (*PR*) gene expression. After an initial local infection, systemic acquired resistance (SAR) often occurs, which induces expression of a set of *PR* genes, leading to a long-lasting enhanced resistance against a broad spectrum of pathogens [1]. In dicots, SA and its synthetic analogs, 2,6-dichloroisonicotinic acid (INA), benzothiadiazole (BTH), and probenazole, are potent inducers of SAR [2–4]. In monocots, SAR is induced by BTH treatment in wheat [5] and by *Pseudomonas syringae* in rice [6]. BTH also induces disease resistance in rice [7–9] and maize [10], although it is unclear if these defense responses are equivalent to SAR.

The *NPR1* (nonexpressor of pathogenesis-related genes 1; also known as *NIM1* and *SAI1*) gene is a key regulator of SA-mediated SAR in Arabidopsis [11–15]. Upon induction by SA, INA, or BTH, *NPR1* expression levels increase, influencing the SAR response [16]. Arabidopsis *npr1* mutants are impaired in their ability to induce *PR* gene expression and cannot mount a SAR response even after treatment with SA or INA. *NPR1* encodes a protein with a bipartite nuclear localization sequence and two protein-protein interaction domains: an ankyrin repeat domain and a BTB/POZ domain [16]. Before activation, NPR1 forms an oligomer that is mostly excluded from the nucleus. Upon SAR induction and subsequent change to the cellular redox state, monomeric NPR1 is released and accumulates in the nucleus, activating *PR* gene expression [17]. It has been hypothesized that NPR1 is an SA receptor that binds directly to SA, resulting in a conformational change that releases its C-terminal transcriptional activation domain and transforms the NPR1 protein into a functional transcriptional co-activator [18]. Another report suggests that NPR3 and NPR4 are the SA receptors carrying higher SA binding affinity than NPR1. In this model, NPR3 and NPR4 binding to SA triggers the Cullin 3 ubiquitin E3 ligase-mediated NPR1 degradation, which is an essential step of NPR1 function [19]. Both these models indicate that SA modulates NPR1 function.

In Arabidopsis, over-expression of *NPR1* leads to enhanced disease resistance against both bacterial and oomycete pathogens [20]. In rice, over-expression of Arabidopsis *NPR1* [21] or the rice ortholog *NH1* [22, 23] results in enhanced resistance to pathogens *Xanthomonas oryzae* pv. *oryzae* (*Xoo*) and *Magnaporthe grisea*. Introduction of an extra copy of the paralogous gene *NH3* in rice also results in enhanced resistance to *Xoo,* as well as hyper-responsiveness to SAR inducer treatment [24].

As previously reported, rice contains five NPR1 homologs (NH) that can be divided into three clades: clade 1 containing NH1 alone, clade 2 comprised of NH2 and NH3, and clade 3 consisting of NH4 and NH5 (with NH5 duplicated in two copies present on chromosomes 11 and 12) [23, 24]. Only NH1 and NH3 enhance resistance to *Xoo* when expressed at elevated levels [22–24]. Thus, two out of the five rice NH proteins are known to be involved in immunity to *Xoo*. NH4 and NH5 are similar to Arabidopsis BOP2 and BOP1 [24], respectively, which are mainly involved in regulating plant development [25]. No evidence suggests that NH2, NH4, and NH5 are involved in plant innate immunity. It is not known how NH1 and NH3 are able to confer a robust immune response while NH2, NH4, and NH5 cannot.

In search for proteins that mediate NPR1 function, several groups have identified NPR1-interaction partners. Using yeast two-hybrid assays, TGA family members of basic-region leucine zipper (bZIP) transcription factors from Arabidopsis [26, 27] and rice [21] have been shown to interact with NPR1. This interaction is mediated by the ankyrin repeats of NPR1 that are necessary and sufficient for this interaction [26]. The interaction between NPR1 and TGA proteins has been demonstrated *in vitro* [27] and *in vivo* [28] to facilitate binding of the TGA proteins to the SA-responsive promoters. *In vivo* interaction between NPR1 and TGA2, fused to the Gal4 DNA binding domain, leads to SA-mediated gene activation in Arabidopsis [29]. The Arabidopsis triple knockout mutant *tga2tga5tga6* blocks SA induction of *PR* gene expression and subsequent pathogen resistance [30]. However, TGA2, TGA5, and TGA6 function redundantly as negative regulators of *PR* genes before induction because their triple mutant leads to higher basal levels of *PR* gene expression [30]. NPR1 functions as a transcriptional co-activator in a TGA2-NPR1 complex after SA treatment in a transient cell assay; this function requires the BTB/POZ domain and the oxidation of NPR1 Cys-521 and Cys-529 [31]. The BTB/POZ domain interacts with the repression domain of TGA2 to neutralize its function [32]. The BTB/POZ domain also serves to sequester and repress the C-terminal transactivation domain of NPR1 and SA induction releases this inhibition [18].

In Arabidopsis, three additional NIM1/NPR1 interacting proteins were identified. These proteins, named NIMIN1, NIMIN2 and NIMIN3, share very limited sequence similarity but all carry an NPR1-interaction domain [33]. Over-expression of *NIMIN1* in Arabidopsis compromises SAR, while knockout and RNA-silencing of *NIMIN1* results in enhanced *PR-1* gene expression after SA treatment, indicating that NIMIN1 is a suppressor of SAR [34]. Similarly, in tobacco, over-expression of *NtNIMIN2* delays *PR-1* induction, while suppression of *NtNIMIN2* enhances the induction of *PR-1* [35]. In rice, we have identified an NH1 interactor: the Negative Regulator of Resistance (NRR) protein. This protein shares limited similarity with Arabidopsis NIMIN1 and NIMIN2 (approximately 20% identity with NIMIN2 and less than 20% with NIMIN1) in the two regions required for interaction with NH1 and for inhibition of NH1-mediated transcriptional activation [36, 37]. Over-expression of NRR results in hyper-susceptibility to *Xoo* and compromises pattern recognition receptor XA21-mediated resistance to *Xoo* in rice [36]. Additionally, over-expression of NRR in Arabidopsis enhances susceptibility to *Pseudomonas syringae* DC3000 by completely blocking SAR induction [38].

To date, the TGA and NRR/NIMIN proteins are the only reported proteins that interact with Arabidopsis NPR1 and rice NH1. We have previously isolated four rice genes encoding TGA family proteins that interact with both Arabidopsis NPR1 and rice NH1 [21]. Expression of a dominant negative form of TGA2.1 in rice leads to slightly higher resistance to *Xoo*, indicating that rTGA2.1 alone may act as a negative regulator to basal immunity in rice [39]. The rice genome contains an additional 11 TGA-like proteins and 3 NRR homologous proteins (termed RH proteins). We hypothesize that these proteins differentially contribute to or regulate NH1- and NH3-mediated immunity. We set out to test this hypothesis by looking at their interaction partners.

Large-scale genome-wide protein-protein interaction surveys have previously been conducted with Arabidopsis immune regulators and microbial effectors and with rice kinases using yeast two-hybrid (Y2H) assays [40–42]. Although very useful for a global view of plant immune responses, these large-scale genome-wide surveys do not address the interaction of the TGA, RH and NH proteins. We hypothesized that a focused survey of the interaction of members of these gene families would reveal preferred partners of NH1 and NH3 and leads to insights concerning how NH1 and NH3 differ from other family members in contributing to rice immunity. We have previously reported the use of a yeast-two hybrid method and a Bimolecular Fluorescence Complementation (BiFC) assay based on split yellow fluorescence protein (YFP) [43, 44] to study the stress response protein interaction network consisting of 100 rice proteins [45]. BiFC has emerged as a key technique to visualize protein-protein interactions *in vivo* in a variety of model organisms [46]. The BiFC assay is based on reconstitution of an intact fluorescent protein when two complementary, non-fluorescent fragments of the fluorescent protein are brought together by a pair of interacting proteins. We also devised a bridged split-YFP assay to test for formation of protein complexes containing a member of each of the three families in protoplasts. Here we applied these two approaches to all members of the NH, RH, and TGA families to assess their interactions on a genome-wide scale. We found strong preferences in interaction partners for NH1 and NH3, which interact more strongly with rTGA2.1, rTGA2.2, rTGA2.3, rLG2, TGAL2 and TGAL4 and RH members than the other NH proteins.

## Results

### NH: TGA interaction profiles demonstrate selectivity in interaction partners

We previously reported the presence of five rice NH proteins that form three clades: NH1 alone in clade 1, NH2 and NH3 in clade 2, and NH4 and NH5 in clade 3 [24]. We have also reported the isolation of four rice TGA transcription factors (rTGA2.1, rTGA2.2, rTGA2.3, and rLG2) that interact with Arabidopsis NPR1 [21] and rice NH1 [22] proteins. We searched available GenBank databases for other rice TGA-like (TGAL) proteins and found 11 genes encoding rice TGAL proteins (TGAL1: gi34909110; TGAL2: gi17025924; TGAL3: gi53793173; TGAL4: gi62732726; TGAL5: gi50931615; TGAL6: gi50929037; TGAL7: gi50899406; TGAL8: gi51091219; TGAL9: gi50905923; TGAL10: gi50941637; TGAL11: gi77553042). We were able to amplify full-length cDNAs for 9 of the 11 TGAL genes (TGAL1 and TGAL2 are alternative transcripts).

To determine which TGA transcription factors are potentially involved in NH1- and NH3-triggered immunity, we investigated interactions between NH proteins and available TGA transcription factors using an Y2H assay. We cloned each rice NH family member into the LexA bait vector and each TGA factor into the B42AD prey vector or vice versus and carried out Y2H tests. Semi-quantitative results based on the relative strength of β-galactosidase reporter activity, ranging from dark blue (marked +++) to white (−), resulting from protein-protein interaction are summarized in Tables 1 and 2. The original pictures of the yeast two-hybrid test results are provided in Additional file 1: Figures S1A and B. We tested 65 reciprocal combinations of NH and TGA proteins, yielding 62 and 48 positive interactions (blue colors). NH proteins interacted with all TGA proteins, with the exception of TGAL9, which interacted with only NH4 and NH5. When fused to LexA, individually, NH1, NH2, and NH3 interacted well with most TGA members except TGAL5, TGAL7, TGA8, and TGA9; the LexA-NH3 fusion also failed to interact with TGAL6. NH4 and NH5 LexA fusions interacted with most members of the TGA family, however not as strongly with rTGA2.1, rTGA2.2, and TGAL2, which showed strong interactions with NH1, NH2, and NH3. Notably, NH4 and NH5 both interacted well with TGAL5, TGAL7, TGAL8, and TGAL9—TGA family members that failed to interact with LexA-NH1, NH2, or NH3 fusions. Thus, interactions between NH and TGA family members are, in certain cases, mutually exclusive. Weak or negative interaction between TGA and NH proteins was unlikely a result of protein instability, as each NH or TGA fusion protein resulted in at least one strong interaction, indicating that all fusions are stable in yeast.

**Table 1:** **Summary of yeast two-hybrid results**

	B42AD fusion					
LexA fusion	NH1	NH2	NH3	NH4	NH5	B42AD
**rTGA2.1**	**+++**	**++**	**+++**	**+++**	**+**	**–**
**rTGA2.2**	**+++**	**++**	**+++**	**+++**	**++**	**–**
**rTGA2.3**	**+++**	**++**	**+++**	**+++**	**+++**	**–**
**rLG2**	**+++**	**++**	**+++**	**+++**	**+++**	**–**
**TGAL1**	**+++**	**+++**	**+++**	**+++**	**+++**	**–**
**TGAL2**	**+++**	**+++**	**+++**	**+++**	**+**	**–**
**TGAL4**	**+++**	**+++**	**+++**	**+++**	**+++**	**–**
**TGAL5**	**+++**	**+++**	**+++**	**+++**	**+++**	**–**
**TGAL6**	**+++**	**++**	**+++**	**+++**	**++**	**–**
**TGAL7**	**++**	**+++**	**+++**	**+++**	**+++**	**+**
**TGAL8**	**+++**	**+++**	**+++**	**+++**	**+++**	**–**
**TGAL9**	**±**	**±**	**±**	**+++**	**+++**	**–**
**TGAL11**	**+++**	**++**	**+++**	**+++**	**+++**	**–**
**LexA**	**–**	**–**	**+**	**+**	**+**	

Rice TGA proteins are fused to LexA and NH proteins fused to B42AD. Protein interactions between the two families were tested and semi-quantitative results recorded. At least three independent yeast colonies were included for each pair of proteins. Strong interactions as judged by dark blue colors developed from X-gal are shown as” +++” and no interactions as judged by white colors indicated as “−”. A subscript “+” indicates a slightly lighter blue color than a regular “+”.

**Table 2:** **Summary of yeast two-hybrid results**

	B42AD fusion													
LexA fusion	B42AD	rTGA2.1	rTGA2.2	rTGA2.3	rLG2	TGAL1	TGAL2	TGAL4	TGAL5	TGAL6	TGAL7	TGAL8	TGAL9	TGAL11
**NH1**	**–**	**+++**	**+++**	**+++**	**+++**	**++**	**+++**	**+++**	**–**	**+++**	**–**	**–**	**–**	**+++**
**NH2**	**–**	**+++**	**+++**	**+++**	**+++**	**+++**	**+++**	**+++**	**–**	**+++**	**–**	**–**	**–**	**+++**
**NH3**	**–**	**+++**	**+++**	**+++**	**+++**	**+++**	**+++**	**+++**	**±**	**–**	**–**	**–**	**–**	**+++**
**NH4**	**+**	**+**	**++**	**+++**	**++**	**++**	**+**	**+++**	**+++**	**++**	**++**	**+++**	**++**	**+++**
**NH5**	**±**	**±**	**++**	**++**	**+++**	**++**	**±**	**+++**	**+++**	**+++**	**++**	**++**	**+++**	**+++**
**LexA**	**–**	**–**	**–**	**–**	**–**	**–**	**–**	**–**	**±**	**–**	**–**	**–**	**–**	**–**

Rice NH proteins are fused to LexA and TGA proteins fused to B42AD. Other criteria are as described in Table 1.

To test whether these interactions held true *in planta*, we carried out split YFP tests in rice protoplasts with both YFPC- and YFPN-NH and TGA fusions. Depending on which portion of YFP was fused to NH or TGA, either 49 or 31 out of 65 tests yielded positive interactions. The results are summarized in Tables 3 and 4 and the original fluorescence images provided in Additional file 2: Figures S2A and B. In fusion with either YFPC or YFPN, NH1 interacted with all TGA members, but preferred rTGA2.1, rTGA2.2, rTGA2.3, rLG2, TGAL2 and TGAL4. NH2 showed strong interactions with TGAL1, TGAL2, and TGAL11 and very weak interactions with rTGA2.2, rTGA2.3 and TGAL7, only when fused to YFPN. NH3 showed a similar TGA interaction pattern as NH1, with preferences for rTGA2.1, rTGA2.2, rTGA2.3, rLG2, TGAL2 and TGAL4. Notably, even though interactions between NH3-rTGA2.3, NH3-rLG2, NH3-TGAL2, and NH3-TGAL4 gave YFP signals of similar intensity as NH1 interactions, consistently fewer cells showed signals (see Additional file 2: Figure S2), indicating that interactions may only occur in certain cell types. NH4-TGA interaction patterns were similar to NH1 and NH3, but with much weaker YFP signals and less obvious preferences for TGA partners. NH5 fused to YFPN or YFPC showed only weak interactions with some TGA members. These results suggest that protein interaction partners are more selective in the split YFP assay in rice cells than in yeast.

**Table 3:** **Summary of split YFP results**

YFPC fusion	YFPN fusion													
	YFPN	rTGA2.1	rTGA2.2	rTGA2.3	rLG2	TGAL1	TGAL2	TGAL4	TGAL5	TGAL6	TGAL7	TGAL8	TGAL9	TGAL11
**NH1**	**±**	**+++**	**+++**	**+++**	**+++**	**++**_**+**_	**+++**	**+++**	**++**	**++**	**+**_**+**_	**+**_**+**_	**+**_**+**_	**++**
**NH2**	**–**	**–**	**±**	**–**	**–**	**–**	**–**	**–**	**–**	**–**	**–**	**–**	**–**	**–**
**NH3**	**–**	**+++**	**++**_**+**_	**+++** ^**a**^	**+++** ^**a**^	**++**	**+++** ^**a**^	**+++** ^**a**^	**++**	**++**	**+**	**+**	**±**	**++**
**NH4**	**–**	**++**	**++**	**++**	**++**	**++**	**++**	**++**	**++**	**++**	**+**_**+**_	**+**_**+**_	**+**_**+**_	**++**
**NH5**	**–**	**+**_**+**_	**+**_**+**_	**+**_**+**_	**++**	**+**	**+**_**+**_	**+**_**+**_	**+**_**+**_	**+**	**+**	**±**	**+**	**–**
**YFPC**		**+**	**+**	**+**	**+**	**±**	**–**	**±**	**–**	**±**	**–**	**–**	**–**	**–**

Rice TGA proteins are fused to YFPN and NH proteins fused to YFPC. Interactions between the two families were tested and semi-quantitative results recorded. Strong interactions as judged by bright YFP fluorescence are shown as” +++” and no interactions as judged by lack of YFP fluorescence indicated as “−”. “^a^” indicates that signals showed in fewer cells compared to other combinations. A subscript “+” indicates a slightly weaker YFP signal than a regular “+”.

**Table 4:** **Summary of split YFP results**

YFPN fusion	YFPC fusion													
	YFPC	rTGA2.1	rTGA2.2	rTGA2.3	rLG2	TGAL1	TGAL2	TGAL4	TGAL5	TGAL6	TGAL7	TGAL8	TGAL9	TGAL11
**NH1**	**–**	**+++**	**+++**	**+++**	**++**	**+**	**+++**	**+++**	**±**	**–**	**–**	**–**	**–**	**+**
**NH2**	**–**	**–**	**+**	**+**	**–**	**++**_**+**_	**+++**	**–**	**–**	**–**	**+**	**±**	**–**	**+++**
**NH3**	**–**	**++**	**++**	**++**	**+**	**+**	**+++**	**+++**	**+**	**–**	**–**	**–**	**–**	**–**
**NH4**	**–**	**+**	**+**	**+**	**–**	**±**	**++**	**++**	**+**	**–**	**–**	**–**	**+**	**–**
**NH5**	**–**	**+**	**+**	**–**	**–**	**–**	**–**	**–**	**–**	**–**	**–**	**–**	**–**	**–**
**YFPN**	**–**	**–**	**–**	**–**	**–**	**–**	**–**	**–**	**–**	**–**	**–**	**–**	**–**	**–**

Rice NH proteins are fused to YFPN and TGA proteins fused to YFPC. Interactions between the two families were tested. Other criteria are as described in Table 3.

**Figure 1:**
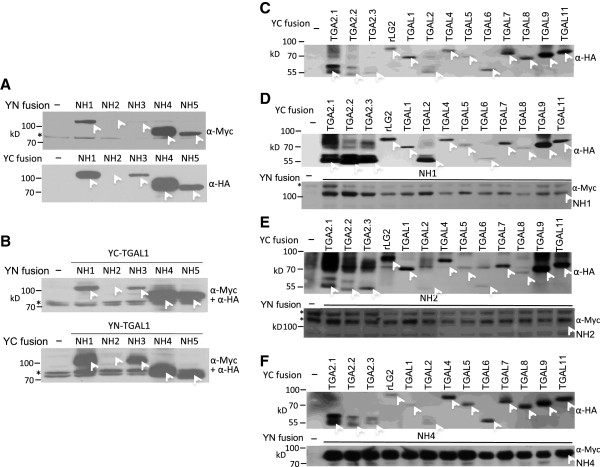
**Western blot analyses of rice NH and TGA proteins fused to YFPN or YFPC and expressed in protoplasts.** **(A)** and **(B)**: Constructs encoding YFPN (YN)-NH1, −NH2, −NH3, −NH4, and –NH5, or YFPC (YC)-NH1, −NH2, −NH3, −NH4, and –NH5 were used to transfect rice protoplasts. A Ubi-Gus plasmid was included as an internal control for transfection. Protoplast cells were harvest 24 hours after transfection. Each protein sample was suspended in 1× SDS loading buffer and amount of loading was adjusted based on GUS activity. The negative control contained plasmids pSY736, carrying YN, and pSY735, carrying YC. **(A)** Western blot analyses were carried out using an α-Myc or an α-HA tag monoclonal antibody. **(B)** Transfections of protoplasts were carried out individually with an YN-NH plasmid together with an YC-TGAL1 plasmid, or an YC-NH plasmid with an YN-TGAL1 plasmid. The negative control sample was the same as above. The nitrocellulose membranes were probed with the α-Myc and α-HA antibodies simultaneously. YC-fused TGA2.1, TGA2.2, TGA2.3, rLG2, TGAL1, TGAL2, TGAL4, TGAL5, TGAL6, TGAL7, TGAL8, TGAL9, and TGAL11 were expressed in protoplasts alone in **(C)**, or with YN-NH1 in **(D)**, YN-NH2 in **(E)**, or YN-NH4 in **(F)**. YC fusions were probed with α-HA and YN-fusions probed with α-Myc antibodies. Molecular weight: YFPN = 22.0kD; YFPC = 14.5kD; NH1 = 63.9kD; NH2 = 67.4kD; NH3 = 65.0kD; NH4 = 53.3kD; NH5 = 51.8kD; TGA2.1 = 37.2kD; TGA2.2 = 37.1kD; TGA2.3 = 36.8kD; rLG2 = 55.7kD; TGAL1 = 51.3kD; TGAL2 = 36.9kD; TGAL4 = 52.9kD; TGAL5 = 48.7kD; TGAL6 = 40.6kD; TGAL7 = 51.3kD; TGAL8 = 49.3kD; TGAL9 = 48.1kD; and TGAL11 = 52.9kD. Each protein is marked with a white arrowhead. The black “*” symbol indicates nonspecific bands detected by the α-Myc antibody.

**Figure 2:**
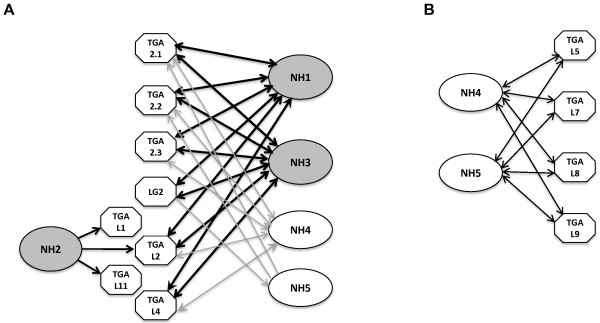
**Interaction network between the rice NH family members and select TGA proteins.** The summary is based primarily on split YFP results with Y2H results taken into consideration. **(A)** Among the five rice NH proteins, NH1 and NH3 are the major interactors of the TGA proteins represented here. Strong interactions are shown as solid dark lines and weak interactions are shown as grey lines. Bidirectional arrows indicate that both YFPN and YFPC fusions interact well, while single-direction arrows indicate that only one of the fusions shows interaction in split YFP. **(B)** TGA interactors unique to NH4 and NH5. Presented are four TGA proteins that interact strongly with NH4 and NH5 in both LexA and B42AD fusions. When fused to B42AD, these TGA proteins fail to interact strongly with NH1, NH2, and NH3. TGAL9 also fails to interacts with NH1, NH2, or NH3 in the LexA fusion.

Because YFPC-NH2 showed no interactions with any of the TGA proteins and YFPN-NH2 showed interactions with few TGA proteins, we carried out Western blot analyses to test if the NH2 fusion proteins were unstable under these conditions. The YFPN (YN) fusion proteins were tagged with a c-Myc epitope and hence probed with an α-c-Myc antibody; the YFPC (YC) fusion proteins were tagged with a hemaglutinin (HA) epitope and thus probed with an α-HA antibody. The amount of protein loaded in each lane was normalized to the β-glucuronidase (GUS) activity expressed from plasmid Ubi-Gus, which was included as an internal control for protoplast transfection. Figures 1A and B show the Western analysis results of the NH1, NH2, NH3, NH4, and NH5 fusion proteins. Each protein is marked with a white arrowhead. In Figure 1A, the NH fusion proteins were expressed alone; in Figure 1B, each of the NH fusion proteins was co-expressed with a YFPN-TGAL1 or a YFPC-TGAL1 protein. In both Figures 1A and B, the NH1, NH4, and NH5 proteins were stably expressed as both YN and YC fusions. The NH3 protein accumulated to a lesser extent, but was readily detectable under most tested conditions. The NH2 fusion proteins were barely detectable under all conditions. The anti-HA antibody in general gave stronger signals than the anti-Myc antibody, most likely due to the differences between these two antibodies. The anti-Myc antibody also detected non-specific bands (marked by a black * sign). These results demonstrate that the NH1, NH3, NH4, and NH5 fusion proteins are stable *in vivo* under the tested conditions. While the NH2 fusion proteins were less stable, the YN:NH2 protein was stable enough to interact with TGAL1, TGAL2, and TGAL11, leading to strong YFP signals. Therefore, even though the low NH2 protein levels may reduce YFP signals, the results of weak or absent signals from interactions between other TGA and NH2 fusion proteins are unlikely solely due to the absence of the NH2 fusion proteins.The expression levels of YFPC fused TGA proteins alone or together with YFPN-NH1, −NH2, or -NH4, were examined by Western analyses and are shown in Figures 1C, D, E, and F. Each protein is marked with a white arrowhead. The results suggest that the TGA proteins are all relatively stably expressed, despite variations among different TGAs. The YFP signal strengths resulted from interactions with TGA proteins are not correlated with the TGA protein levels, indicating that the expression level of a TGA protein is not the major factor determining the YFP signal strength. Notably, TGA2.1, TGA2.2, TGA2.3, and TGAL2 showed double bands near the locations of the expected molecular weights, suggesting protein modifications. However, it remains unclear as to what modifications may have caused the generation of the multiple forms. In addition, most of the TGA proteins showed additional bands at locations of much higher molecular weights; these bands likely represent dimers of each protein even though each protein was subject to standard procedures of denaturation using SDS and reducing agent before running SDS gels. The expression levels of YFPN-NH1, −NH2, and -NH4 are consistent with previous observations: NH1 and NH4 fusion proteins are stable while NH2 fusion protein is unstable and appears at a very low level.Figure 2 presents an interaction network of rice NH and TGA proteins, based primarily on the split YFP results, but with yeast two-hybrid results taken into consideration. Members of the TGA and NH protein families demonstrated preferences in interaction partners, prompting the classification of NH proteins into two main subfamilies. Figure 2A focuses on major TGA interactors of NH1, NH2, and NH3 (depicted by black arrows). These TGA proteins showed lower preferences for NH4 and even lower preferences for NH5 (grey arrows). NH2 showed similar interaction preferences as NH1 in yeast two-hybrid, but failed to show strong interactions in split YFP for most TGA proteins, except for TGAL1, TGAL2, and TGAL11 when fused to YFPN (depicted as single direction arrows). Figure 2B shows the TGA proteins that preferred NH4 and NH5, instead of NH1, NH2, or NH3. Thus the NH proteins can be roughly divided into two subfamilies based on their interaction patterns with TGA proteins: NH1, NH2, and NH3 in one family and NH4 and NH5 in the other. In summary, the split YFP and Y2H results indicate that NH1 and NH3 are the major interaction partners of rice TGA proteins (depicted as solid dark lines). Interaction profiles also show that NH1 and NH3 prefer rice TGA2.1, TGA2.2, TGA2.3, LG2, TGAL2, and TGAL4 as partners and that NH2 prefers TGAL1, TGAL2, and TGAL11, while NH4 and NH5 have weak or limited interactions (shown as grey lines) with these TGA proteins. NH4 and NH5 may prefer TGAL5, TGAL7, TGAL8, and TGAL9 instead.

### NH1 and NH3 are the preferred interacting partners of RH proteins

Four members of the NRR family in rice have been reported: NRR, RH1, RH2, and RH3 [36]. Because RH proteins potentially play a role in modulating defense, we tested interactions between NH proteins and RH proteins in Y2H and split YFP. Tables 5 and 6 summarize the yeast two-hybrid results and the original pictures are provided in Additional file 3: Figures S3A and B. The Y2H assay on NH-RH interactions yielded either 16 or 18 positives out of 20 combinations, depending on which vector was used. When fused to LexA, NH1, NH2, and NH3 interacted strongly with NRR, RH1, and RH3, but only weakly with RH2. When fused to B42AD, NH1, NH2, and NH3 interacted with all NRR members, albeit less strongly with RH2. NH4 and NH5 in fusion with LexA showed only weak interactions with NRR and RH3, whereas in B42AD fusion, NH4 and NH5 showed interactions with RH1, RH2, and RH3, but not with NRR. These results show that NRR, RH1, and RH3 are the preferred partners of NH1, NH2 and NH3.

**Table 5:** **Summary of yeast two-hybrid results**

	YFPC fusion				
LexA fusion	B42AD	NRR	RH1	RH2	RH3
**NH1**	**–**	**+++**	**+++**	**±**	**+++**
**NH2**	**–**	**+++**	**+++**	**+**	**+++**
**NH3**	**±**	**+++**	**+++**	**+**	**+++**
**NH4**	**+**	**++**	**+**	**++**	**++**
**NH5**	**±**	**++**	**±**	**±**	**++**
**LexA**	**–**	**±**	**–**	**–**	**–**

Rice NH proteins are fused to LexA and RH proteins fused to B42AD. Other criteria are as described in Table 1.

**Table 6:** **Summary of yeast two-hybrid results**

	B42AD fusion					
LexA fusion	NH1	NH2	NH3	NH4	NH5	B42AD
**NRR**	**+++**	**+++**	**+++**	**–**	**–**	**–**
**RH1**	**+++**	**+++**	**+++**	**+++**	**+++**	**–**
**RH2**	**++**	**++**	**++**_**+**_	**+++**	**++**_**+**_	**–**
**RH3**	**+++**	**++**_**+**_	**+++**	**++**	**++**	**–**

Rice RH proteins are fused to LexA and NH proteins fused to B42AD. Other criteria are as described in Table 1.

**Figure 3:**
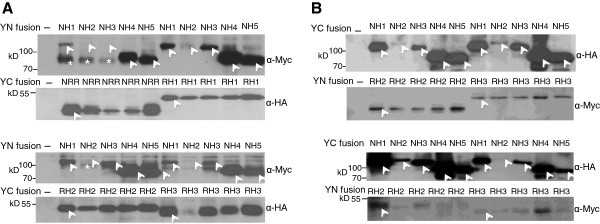
**Western blot analyses of YN- or YC-fused NH proteins and RH proteins in protoplasts.** **(A)** Rice protoplasts were transfected individually with an YN-NH plasmid plus an YC-RH plasmid as described in Figure 1. The white “*” symbols indicate cleaved products of NH1, NH2, and NH3, respectively. **(B)** Protoplasts were transfected with an YC-NH plasmid and an YN-RH plasmid. The negative control contained plasmids pSY736 and pSY735 encoding YN and YC respectively. Protein samples were prepared as in Figure 1 and gel loading was normalized to GUS activity. The NC membranes were probed with α-Myc then α-HA, or α-HA then α-Myc, sequentially. Molecular weight: NRR = 14.2kD; RH1 = 19.2kD; RH2 = 18.1kD; and RH3 = 18.3kD. Molecular weights of YFPN, YFPC, NH1, NH2, NH3, NH4, and NH5 are listed in Figure 1. Each protein is marked with a white arrowhead.

**Figure 4:**
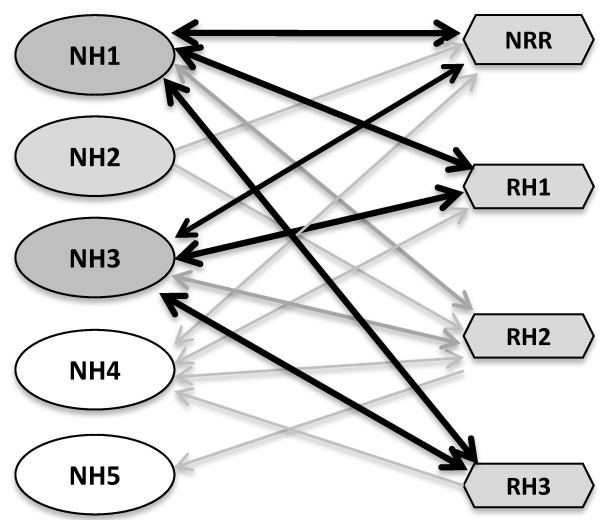
**Interaction network between rice NH and RH family proteins.** The summary is based primarily on split YFP results with Y2H results taken into consideration. Among the five rice NH proteins, NH1 and NH3 are the major interactors of RH proteins. NRR, RH1, and RH3 are the major interactors of NH1 and NH3. Strong interactions are shown as solid dark lines and weak interactions are shown as grey lines. Bidirectional arrows indicate that both YFPN and YFPC fusions interact well, while single arrows indicate that only one of the fusions interacts in split YFP.

Tables 7 and 8 summarize the semi-quantitative spilt YFP results of interactions between NH and RH proteins. The original YFP fluorescence images are provided in Additional file 4: Figures S4A and B. Depending on which portion of YFP was fused to NH or RH family members, either 16 or 14 out of 20 combinations yielded positive interactions above control backgrounds (Table 7 and 8). Whether fused to YFPN or YFPC, NH1 and NH3 behaved similarly, interacting well with NRR, RH1, and RH3, and to a lesser extent with RH2. NH2 interacted weakly with NRR and RH2 when fused to YFPN. Significant background YFP fluorescence signals were observed when NRR and RH1 were fused to YFPN. NH4 and NH5 showed very weak or absent interactions with RH proteins, indicating that NH4 and NH5 may not be the preferred partners of the RH proteins.We conducted Western blot analyses on NH proteins fused either to YFPN (YN) or YFPC (YC) in the presence of a RH protein and on RH proteins fused to the other half of the YFP protein. The results are shown in Figures 3A and B, where each protein is marked with an arrowhead. Overall the results are similar to those presented in Figure 1. NH1, NH4 and NH5 were highly expressed; YN–NH3 was easily detectable, but at lower levels; while YN-NH2 was expressed at the lowest level and was barely detectable in many cases. In general, NRR, RH1, RH2, and RH3 were all stably expressed. Protein loadings were equalized to the GUS activity, expressed from a Ubi-Gus plasmid included as an internal control during protoplast transfection. Notably, YN-NH1, −NH2, and –NH3 fusion proteins (but not YN-NH4 or YN-NH5) were cleaved and appeared mainly at lower molecular weights (marked with a white star in Figure 3A) in the presence of YC-NRR; the reason of this cleavage is unknown.Figure 4 presents an interaction network of the NH and RH proteins, mainly based on split YFP results. NH1 and NH3 are the major partners of all RH proteins and interact strongly (depicted as solid dark lines) with all RH proteins, except RH2 (grey lines). NH2, NH4, and NH5 only have minor interactions (grey lines) with select RH proteins.

**Table 7:** **Summary of split YFP results**

	YFPC fusion				
YFPN fusion	YFPC	NRR	RH1	RH2	RH3
**NH1**	**+**	**+++**	**+++**	**++**_**+**_	**+++**
**NH2**	**–**	**++**	**–**	**++**	**–**
**NH3**	**+**	**+++**	**+++**	**++**_**+**_	**+++**
**NH4**	**+**	**++**	**++**	**++**	**±**
**NH5**	**+**	**++**	**++**	**++**	**±**
**YFPN**		**+**	**+**	**+**	**±**

Rice NH proteins are fused to YFPN and TGA proteins fused to YFPC. Interactions between the two families were tested. Other criteria are as described in Table 3.

**Table 8:** **Summary of split YFP results**

	YFPC fusion					
YFPN fusion	YFPC	NH1	NH2	NH3	NH4	NH5
**NRR**	**+**_**+**_	**+++**	**+**	**++**_**+**_	**++**	**+**
**RH1**	**+**_**+**_	**+++**	**+**	**+++**	**++**	**++**
**RH2**	**+**	**++**_**+**_	**–**	**++**_**+**_	**++**	**++**
**RH3**	**+**	**+++**	**+**	**+++**	**+**_**+**_	**±**
**YFPN**		**±**	**–**	**±**	**±**	**–**

Rice RH proteins are fused to YFPN and NH proteins fused to YFPC. Interactions between the two families were tested. Other criteria are as described in Table 3.

### A bridged split YFP assay confirms that NH1, NH2, and NH3 form complexes containing specific RH and TGA proteins

The experiments described above suggest a broad and general capacity for interaction between members of the three families of proteins that are key regulators of the rice immune response. They also indicate that particular family members preferentially interact with particular subclasses of proteins. These experiments yield valuable information concerning interactions when proteins from two families are present. In order to further investigate the preferred interaction partners when all three families of proteins are present, we devised a bridged split YFP assay. No available evidence suggests that TGA and RH (or NIMIN) proteins directly interact with each other, while both interact with NH proteins, as shown above. Using YFPC-fused RH proteins and YFPN fusions of the TGA proteins determined to be the major interaction partners of NH proteins from the split YFP assay (rTGA2.1, rTGA2.2, rTGA2.3, rLG2, TGAL2, and TGAL4), we tested whether the introduction of various NH proteins (non-YFP fusions) was able to facilitate interaction between YFPN and YFPC fusions, yielding a fluorescence signal.

Table 9 summarizes the semi-quantitative results of the bridged split YFP assay. The original fluorescence images are provided in Additional file 5: Figure S5. When YFPC control and YFPN-fused TGA proteins were expressed together with NH1, NH2, NH3, NH4, or NH5 (expressed from the maize *Ubi-1* promoter and labeled NH1ox, NH2ox, NH3ox, NH4ox, and NH5ox), no YFP fluorescence signal was detected. Similarly, when YFPN control and YFPC-fused NRR, RH1, RH2, or RH3 were expressed with ectopic NH1, NH2, NH3, NH4, or NH5 in protoplasts, little to no YFP signal was detected. When YFPC-fused NRR was co-expressed with NH family members and the YFPN-fused TGA members, 15 positive interactions (only signal strengths marked “+” or higher are counted) were detected among 36 pairs of tests. Without the ectopic expression of NH members, only TGAL4 yielded a weak signal, indicating that TGAL4 may be a prevalent indirect partner of NRR. The weak signal probably represents the low levels of endogenous NH members that associate with these two proteins. When NH1 was ectopically introduced, strong YFP signals appeared for all YFPN-fused TGA members investigated. These results indicate that NH1 can form a complex with NRR and any of these TGA proteins. Introduction of NH3 into the NRR-TGA split YFP system yielded results similar to NH1, despite somewhat weaker YFP signals. These results indicate that, like NH1, NH3 can form a complex consisting of NRR and any of the TGA proteins. The slightly lower YFP signal strengths of NH3 than NH1 were possibly due to the lower protein levels of NH3 than NH1, as detected consistently as YFP fusion proteins in Western analysis shown above. However, we cannot rule out the possibility that NH3 may actually have a lower affinity than NH1 for these TGA and NRR proteins. Consistently, much fewer cells displayed YFP signals when NH3 was introduced compared to NH1 (see Additional file 5: Figure S5A), further suggesting that NH3 may be stable only in certain, but not all, cell types. When NH2 was introduced in place of NH1 into the NRR-TGA split YFP system, no obvious effects were observed, but a weak signal increase from rTGA2.3. Introduction of NH4 or NH5 had little effect on the NRR-TGA system, indicating that NH4 and NH5 probably do not form complexes containing NRR and one of these TGA proteins.

**Table 9:** **Summary of bridged split YFP results**

		YFPN fusion						
YFPC fusion		rTGA2.1	rTGA2.2	rTGA2.3	rLG2	TGAL2	TGAL4	YFPN
**YFPC**	**NH1ox**	**–**	**–**	**–**	**–**	**–**	**–**	
	**NH2ox**	**–**	**–**	**–**	**–**	**–**	**–**	
	**NH3ox**	**–**	**–**	**–**	**–**	**–**	**–**	
	**NH4ox**	**–**	**–**	**–**	**–**	**–**	**–**	
	**NH5ox**	**–**	**–**	**–**	**–**	**–**	**–**	
**NRR**	**–**	**–**	**–**	**–**	**–**	**–**	**+**	
	**NH1ox**	**+++**	**+++**	**+++**	**+++**	**+++**	**+++**	**–**
	**NH2ox**	**±**	**±**	**+**	**–**	**±**	**+**	**–**
	**NH3ox**	**++**	**++**	**++**	**++**	**++**	**++**	**±**
	**NH4ox**	**–**	**–**	**–**	**–**	**–**	**+**	**–**
	**NH5ox**	**–**	**–**	**–**	**–**	**–**	**±**	**–**
**RH1**	**–**	**–**	**±**	**±**	**±**	**±**	**+**	
	**NH1ox**	**+++**	**+++**	**+++**	**+++**	**+++**	**+++**	**±**
	**NH2ox**	**±**	**±**	**±**	**–**	**+**	**+**	**–**
	**NH3ox**	**+++**	**+++**	**+++**	**++/+**	**+++**	**+++**	**±**
	**NH4ox**	**±**	**+**	**+**	**±**	**±**	**+**	**–**
	**NH5ox**	**–**	**–**	**±**	**–**	±	**+**	**–**
**RH2**	**–**	**±**	**–**	**–**	**+**	**+**	**+/++**	
	**NH1ox**	**+**	**+**	**++**	**+**	**+++**	**++**	**–**
	**NH2ox**	**++**	**+++**	**+++**	**++**	**+++**	**++**	**–**
	**NH3ox**	**+++**	**++**	**+++**	**+++**	**+++**	**+++**	**–**
	**NH4ox**	**±**	**±**	**±**	**±**	**±**	**±**	**–**
	**NH5ox**	**–**	**–**	**–**	**–**	**–**	**–**	**–**
**RH3**	**–**	**–**	**–**	**–**	**–**	**–**	**–**	
	**NH1ox**	**+++**	**+++**	**+++**	**+++**	**+++**	**+++**	**–**
	**NH2ox**	**+++**	**+++**	**+++**	**+**	**+++**	**–**	**–**
	**NH3ox**	**–**	**–**	**+**	**–**	**±**	**+**	**–**
	**NH4ox**	**–**	**–**	**–**	**–**	**–**	**–**	**–**
	**NH5ox**	**–**	**–**	**–**	**–**	**–**	**–**	**–**

Rice TGA proteins are fused to YFPN and RH proteins fused to YFPC. Each NH protein was co-introduced with the YFPN and YFPC fusion proteins into protoplasts. Indirect interaction between a TGA and a RH protein, bridged by a NH protein, reconstitutes YFP fluorescence. Semi-quantitative results were recorded. Bright YFP fluorescence are shown as” +++” and lack of YFP fluorescence indicated as “−”.

When YFPC-fused RH1 and select YFPN-fused TGA proteins were co-introduced with NH family members into rice protoplasts, 19 positives were detected out of 36 tests. In the absence of NH proteins, modest YFP signals were observed for YFPN-TGAL4 while no significant signals were detected for other TGA proteins, indicating that TGAL4 is also a prevalent indirect partner for RH1. When NH1 was ectopically introduced, strong YFP signals were detected with all TGA members, indicating that NH1 can facilitate complex formation with RH1 and any of these TGA proteins. Introduction of NH3 had an effect similar to the addition of NH1, suggesting that NH3 can also form a complex consisting of RH1 and any one of the six TGA proteins. Introduction of NH2, NH4, or NH5 had little to no effect on the RH1-TGA split YFP system, suggesting that these proteins do not significantly mediate the formation of complexes containing RH1 and one of the TGA proteins.

When the YFPC-RH2 fusion and select YFPN-fused TGA proteins were co-introduced with NH family members into rice protoplasts, 23 positive signals were detected out of 36 tests. Without ectopic NH proteins, modest signals were observed for rTGA2.1, rTGA2.3, rLG2, TGAL2, and TGAL4, indicating that RH2 can form a complex containing each of these TGA proteins. Introduction of NH1 strengthened the YFP signal associated with TGAL2, and to a lesser extent, the signals for rTGA2.3 and TGAL4 as well, indicating that NH1 is able to mediate complex formation with RH2 and TGAL2, and possibly between RH2 and rTGA2.3 or TGAL4 in protoplasts. Introduction of NH2 yielded strong signals for all rTGA proteins, especially for rTGA2.2, rTGA2.3, and TGAL2, indicating that NH2 is capable of forming a complex containing RH2 and any one of these TGA proteins. When NH3 was introduced, strong signals were observed for rTGA2.1, rTGA2.3, rLG2, TGAL2 and TGAL4, and lower signals for rTGA2.2, suggesting that NH3 can also form a complex containing RH2 and any of these TGA proteins. Introduction of NH4 and NH5 did not increase the YFP signal for any of the TGA family members tested. Instead, it appeared to decrease the background signal observed for rTGA2.1, rTGA2.3, rLG2, TGAL2, and TGAL4. These results suggest that NH4 and NH5 may interact with RH2 or these TGA proteins, but neither forms a complex containing RH2 and any of these TGA proteins. When NH4 or NH5 was ectopically expressed, it interrupted the interaction between RH2 and rTGA2.1, rTGA2.3, rLG2, TGAL2, or TGAL4, and possibly NH3.

**Figure 5:**
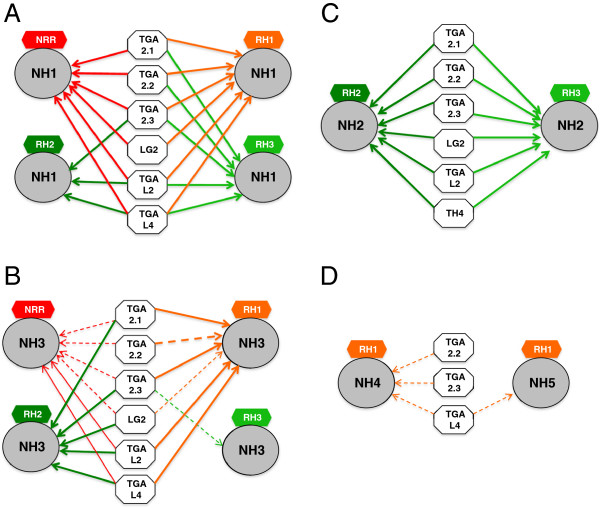
**A schematic representation of TGA, NH, and RH protein complexes.** The array of protein complexes is deduced from the bridged split YFP results. **(A)** NH1-mediated protein complexes. **(B)** NH3- mediated protein complexes. **(C)** NH2- mediated protein complexes. **(D)** NH4- and NH5- mediated protein complexes. The arrows are colored according to the RH protein that associates with the selected NH protein. Solid lines suggest strong associations while dashed lines indicate weak ones.

When YFPC-fused RH3 was co-introduced with YFPN-fused TGA proteins, no YFP signals were detected, suggesting that these proteins do not interact with each other in protoplasts under our experimental conditions. When these combinations were tested with the addition of NH family members, 13 positive interactions were detected from 36 combinations. Ectopic introduction of NH1 resulted in strong YFP signals from all six TGA proteins, suggesting that NH1 can form a complex containing RH3 and any of the six TGA proteins. Introduction of NH2 also led to strong YFP signals from rTGA2.1, rTGA2.2, rTGA2.3, and TGAL2, and a weaker signal from rLG2. Thus, NH2 can form a complex with RH3 and rTGA2.1, rTGA2.2, rTGA2.3, and TGAL2. Introduction of NH3 resulted in weak signal intensity for rTGA2.3 and TGAL4, suggesting that NH3 may form a complex with RH3 and rTGA2.3 or TGAL4, albeit in low abundance. Introduction of NH4 or NH5 had no effect on YFP signals, suggesting that NH4 and NH5 may not form complexes with RH3 and these TGA proteins.Our bridged split YFP data reveals the complexity and specificity of TGA, RH, and NH protein family interactions. A summary of preferred interaction partners deduced from the bridged split YFP experiments is presented in Figure 5. Figure 5A depicts the TGA and RH proteins that associate with NH1 and Figure 5B shows those that associate with NH3. Both NH1 and NH3 interact with rTGA2.1, rTGA2.2, rTGA2.3, rLG2, TGAL2, and TGAL4. NH1 interacts well with all four RH proteins, despite weaker interaction with RH2, and NH3 interacts well with three RH members, not including RH3. Strong interactions are depicted as solid lines and weak ones as dashed lines. Figure 5C presents partners of NH2. NH2 also interacts with the six TGA members tested in protoplasts, but only in the presence of RH2 or RH3. Partners of NH4 and NH5 are depicted in Figure 5D, where NH4 only weakly associates with RH1 and three TGA members: rTGA2.2, rTGA2.3, and TGAL4. NH5 shows weak association with RH1 and TGAL4.

### NH3 is tightly co-expressed with NH1, consistent with their roles in rice defense responses

**Figure 6:**
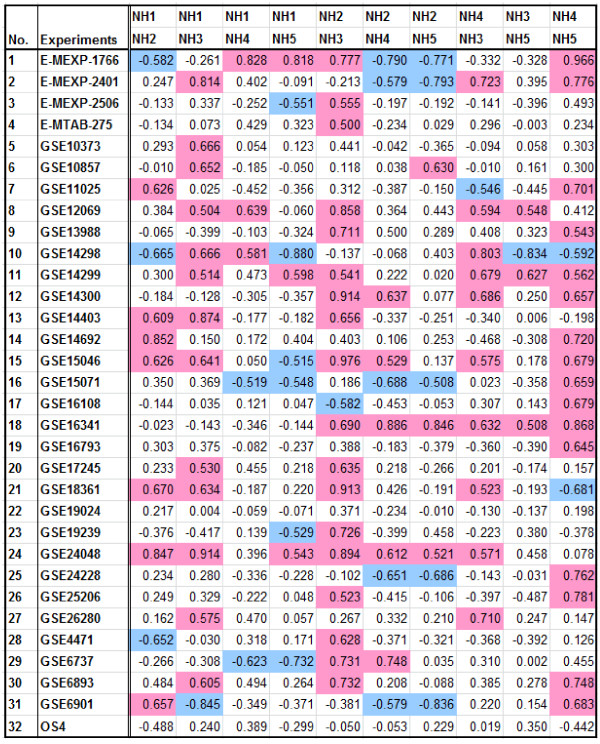
**Expression coefficient of rice NPR1-like members under various experimental conditions.** The expression coefficients equal to or higher than 0.5 are highlighted in purple and those equal to or lower than -0.5 in blue.

Because genes regulating the same pathways are likely to be co-expressed [47, 48], we examined the expression of members of the NH and RH protein families. We analyzed NH1, NH2, NH3, NH4, and NH5 expression levels from 32 publicly available Affymetrix rice microarray data sets covering a broad range of experimental and developmental conditions (conditions listed in Additional file 6: Table S1). We employed a previously reported tool for co-expression analysis [45]. Figure 6 presents the expression coefficient data for each pair of genes. Because NH1 is the most well known member of rice NPR1-like proteins involved in biotic stress responses, we used NH1 as the reference for comparison with other members of the same family. When an expression coefficient of ≥0.5 (highlighted in purple) was set for positive correlation and a coefficient of ≤ −0.5 (highlighted in light blue) was set for inverse correlation, NH2 showed seven positive correlations and three inverse correlations out of the 32 sets of data examined. When the same data sets were probed for NH1 and NH3 expression correlation, however, 13 positive correlations and one inverse correlation were determined, suggesting that the expression of these two genes is strongly linked. In comparison with NH1, NH4 yielded 3 positive correlations and and one inverse correlation, and NH5 had 3 positive correlations and 6 inverse correlations. Thus, among the NPR1-like genes, NH3 expression is most closely associated with NH1 expression.

We also examined whether other members of the NH family demonstrated strong co-expression profiles. When NH3 expression was used as the reference for NH2, 17 positive correlations and one inverse correlation were determined. NH3 and NH4 showed 10 positive correlations and no inverse correlations, while NH5 had 3 positive correlations and only one inverse correlation with NH3 expression. These data suggest that NH2 and NH3 expression are highly correlated, which is consistent with our previous report that NH2 and NH3 are members of the same clade within the NH family [24]. NH3 and NH4 expression are also highly correlated, suggesting that NH3 may also work with NH4 under certain conditions. When NH4 and NH5 expression were compared, 16 positive correlations and only one inverse correlation were detected. These data are consistent with our previous report, which showed that NH4 and NH5 are most closely related to one another and form a single clade and are involved in plant development, rather than biotic stress responses [24].

In summary, our co-expression analyses suggest that among the rice NPR1-like homologs, NH3 expression is most closely correlated with that of NH1, consistent with our previous reports showing that both NH1 and NH3 contribute to rice defense responses. NH3 also shows a high degree of co-expression with NH2 and NH4, demonstrating that it is a versatile protein with a number of potential roles in defense and development.

## NH3 is most highly co-expressed with RH1 and RH2

**Figure 7:**
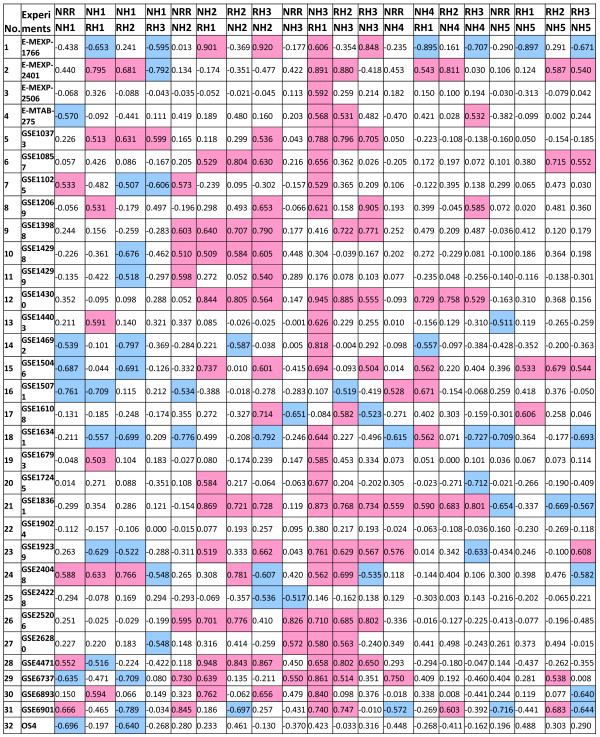
**Expression coefficient between rice NH1 members and NRR members under various experimental conditions.** The expression coefficients equal to or higher than 0.5 are highlighted in purple and those equal to or lower than -0.5 in blue.

Using the aforementioned criteria, we also analyzed the expression coefficients between NH family members and RH family members to assess which members have overlapping expression profiles (Figure 7). Most strikingly, NH3 was most highly co-expressed with RH1, showing 24 positive correlations and no inverse correlations out of the 32 data sets analyzed. NH3 was also highly co-expressed with RH2 and RH3, displaying 14 positive correlations and one inverse correlation and 10 positive correlations and two inverse correlations, respectively. NRR showed little co-expression with NH3, having three positive correlations and two negative ones.

Other members of the NH family also showed a significant overlap in expression with members of the RH family. NH2 showed high co-expression with RH1 (13 positive and no inverse correlations) and RH3 (14 positive and 3 inverse correlations). NH2 also showed modest co-expression with RH2 (8 positive and 2 inverse correlations) and with NRR (7 positive and 2 inverse correlations). NH1, however, showed much lower positive co-expression with NRR, RH1, RH2, and RH3 than either NH2 or NH3, yet NH1 showed a notably higher degree of inverse correlation with these genes (6, 5, 10, and 5 inverse correlations, respectively). Finally, NH4 showed a modest correlation with RH1 (6 positive and 2 inverse correlations), while NH5 displayed a very low degree of correlation with NRR (4 inverse correlations), RH2 (5 positive and one inverse correlations), and RH3 (4 positive and 6 inverse correlations).

These results suggest that of the genes present in these two families, the function of NH3 is the most positively associated with RH1 and RH2. Conversely, NH1 function is the most negatively correlated with RH family members. These expression results represent a major difference between NH1 and NH3 expression patterns and suggest that the RH proteins may differentially modulate NH1 and NH3 functions under different conditions.

We also examined co-expression patterns between NH and rice TGA genes. However, no clear trends were observed and thus are not shown or discussed here.

## Discussion

Yeast two hybrid assays have been widely used to investigate interaction networks among a large number of proteins [40, 41]. For example, Mukhtar et al. surveyed approximately 8552 proteins and found 1358 interactions among 926 proteins, including 83 microbial effectors, 170 immune proteins, and 673 other Arabidopsis proteins [40]. One drawback to large-scale Y2H assays, however, is that due to the possible occurrence of false positive interactions, independent *in vivo* approaches are needed to validate their biological relevance. For example, the validation rates of Y2H-based interactions described on the Arabidopsis Interactome version 1 main screen were about 80% of the original number identified [41]. However, it is usually impossible to verify all of the results with another independent approach, such as co-immunoprecipitation, considering the amount of labor involved. Yet efforts towards this end have been made; a rice kinase protein interaction map containing 116 representative rice kinases and 254 of their interacting proteins was previously reported [42]. In this study, the rate of overlapping interactions identified by Y2H and by TAP tag (tandem affinity purification tag)-based co-immunoprecipitation coupled with mass-spectrometry was low—only four interactors overlapped among the 254 identified by the two methods [42]. This low rate of overlap is not unique to this study. Similarly, a low (7%) rate of overlap was found in the yeast proteome when Y2H data sets were compared with protein complex data sets generated by TAP tag-based co-immunoprecipitation [49]. In contrast, the results from our split YFP and Y2H experiments indicate that they generally agree, with overlapping interaction rates between 70% and 90%, rates considerably higher than those observed when co-immunoprecipitation experiments are used for validating Y2H interactions. In general, split YFP assays in protoplasts reveal a higher selectivity for interaction partners than the Y2H assay and are an efficient *in vivo* method to verify Y2H results.

For the two halves of split YFP to reconstitute a functional YFP and give fluorescence signals, the two fusion proteins need to be expressed and accumulate in the same cell types and same cellular compartments. The YFPN and YFPC polypeptides need to be proximal to each other enough to form a functional YFP protein. These requirements are not needed for yeast two hybrid assays to work. Thus, in theory and in practice, split YFP assays integrate cellular conditions and are more stringent than yeast two-hybrid assays. Our conclusions are mainly drawn from split YFP results. The discrepancies that occur when different halves of YFP were fused to the protein may be due to interferences when YFPN or YFPC was fused to the test protein, leading to false negatives. In view of this, positive results may be taken over negative results in split YFP assays.

Our survey of the protein-protein interactions between rice NH, TGA, and RH families reveals a broad, complex interaction network among these three families of proteins and allows us to draw important conclusions. First of all, each rice NH protein can partner with several different rice TGA and RH proteins. Second, these NH proteins show distinct preferences in interaction partners, indicating that while complexes formed by these proteins appear heterogeneous in nature, certain combinations of NH, RH, and TGA proteins are more likely to occur than others. And finally, members of the NH family cluster according to their interaction profiles with different TGA and RH proteins. NH1, NH2, and NH3 form a cluster, while NH4 and NH5 form another cluster. Within the NH1, NH2 and NH3 cluster, NH1 and NH3 interaction profiles are more closely related to each other with regard to their abilities to interact with rice TGA and RH proteins. These results are consistent with our previous finding that NH1 and NH3 are the only members of the rice NPR1-like protein family to enhance resistance to *Xoo* [24]. Other rice NH proteins failed to show positive regulatory effects on immune responses [23].

The immune response-enhancing effects of NH1 and NH3 may be associated with their ability to interact strongly with the same subgroup of rice TGA proteins: rTGA2.1, rTGA2.2, rTGA2.3, rLG2, TGAL2, and TGAL4. Thus, our study on the interaction network of the three families of innate immune regulators may have revealed an important clue as to why NH1 and NH3 can regulate immune responses to *Xoo*, whereas the other NH proteins do not. Moreover, this group of TGA transcription factors may have emerged as important players in immunity. We hypothesize that NH1 and NH3 bind to these TGA transcription factors and act as transcriptional co-activators. One approach to test this hypothesis is to assess the ability of NH1 and NH3 to activate expression of a reporter gene in the presence of rTGA2.1, rTGA2.2, rTGA2.3, rLG2, TGAL2, or TGAL4, but not the other TGA factors.

Results of the bridged split YFP assay are generally consistent with the direct split YFP results, but appear more selective. For instance, NH1 and NH3 interact strongly with the same six TGA proteins in both the direct and bridged split YFP assay. NH1 and NH3 also interact with the four RH proteins to varying degrees in both the direct and bridged split YFP assays. However, discrepancies do exist. Some combinations of RH and TGA proteins appear to disrupt interactions observed in the direct split YFP assay between NH proteins and members of these other two families. For example, NH3 interacts well with RH3 and all six selected TGA proteins in the direct split YFP assay, but not in the bridged split YFP assay, where only rTGA2.3 and TGAL4 show a weak association with NH3 in the presence of ectopic RH3. This might be due to steric hindrance resulting from the NH3:RH3 interaction, excluding rTGA proteins from binding to the complex, or the vice versa. NH2 did not show strong interaction with TGA proteins in the direct split YFP assay (Table 3), but does associate strongly with the six selected TGA proteins in the presence of ectopic RH2 or RH3 (Table 9). One possibility is that association of NH2 with RH2 or RH3 may stabilize the NH2 protein, allowing NH2 to accumulate and partner with TGA proteins to produce stronger YFP signals in bridged YFP experiments. However, under normal conditions, RH2 and RH3 proteins may not be present at levels high enough to have such an obvious effect.

NH4 and NH5 also showed differences between interaction profiles depending on whether a direct or bridged split YFP assay was used. In direct split YFP assays, NH4 and NH5 interacted modestly with most rice TGA and RH proteins with few outstanding preferences. However, in the bridged split YFP assay, only rTGA2.2, rTGA2.3, and TGAL4 associated weakly with NH4 in the presence of RH1. Similarly, only TGAL4 and RH1 shared a weak association when NH5 was present. Formation of protein complexes in the bridged split YFP assay appeared to be more selective. The observation that few members of the subset of TGA and RH proteins tested could form complexes with NH4 and NH5 may also contribute to the fact NH4 and NH5 are not involved in innate immunity to *Xoo*. Our Y2H data supports this possible conclusion, in that NH4 and NH5 (orthologs of Arabidopsis BOP1 and BOP2, *blade-on-patiole 1 and blade-on-patiole*2) interacted strongly with the TGAL proteins that failed to interact with NH1 and NH3: TGAL5, TGAL7, TGAL8, and TGAL9 (Table 2). These results indicate that these TGA proteins may be involved in plant development rather than defense, similar to Arabidopsis BOP1 and BOP2. This hypothesis can be tested via silencing of these TGAL genes. However, silencing of multiple TGAL genes may be necessary to observe clear phenotypes. Because these TGALs likely mediate the functions of NH4 and NH5, we hypothesize that *bop*-like developmental phenotypes [50, 51] would appear in the silenced lines. The BOP genes have also been suggested to be involved in methyl jasmonate-mediated resistance in Arabidopsis [52], a defense response distinct from the SA-mediated immune response [53]. These results suggest that silencing of these *TGAL* genes whose products interact with NH4 and NH5 may block jasmonate-induced defense response in rice.

In Arabidopsis, TGA2, TGA5, and TGA6 appear to be functionally redundant. Alterations in NPR1-mediated SAR response after inducer treatment can only be observed in plants knocked out for all three genes [30]. The rice genome encodes at least 15 TGA-like proteins and some of them may also function redundantly. Our protein-protein interaction results point to rTGA2.1, rTGA2.2, rTGA2.3, rLG2, TGAL2, and TGAL4 as the important candidates for mediating NH1 and NH3 function. Silencing of these six TGA-encoding genes singly or multiply will help elucidate the involvement of these TGA proteins in the rice immune response.

Finally, co-expression analyses of 32 sets of rice microarray data lead us to the conclusion that of all NH members, NH3 expression most closely parallels that of NH1, consistent with the above observation that they interact with similar proteins and share common roles in plant immunity. RH1 expression is most positively correlated with NH3 expression, strongly suggesting that these two proteins may function together. This notion is consistent with the Y2H and split YFP results that show that these two proteins interact strongly with each other—a relationship that is not observed for RH1 and NH1. It remains to be determined what role this NH3-RH1 relationship plays in plants. On the other hand, NH1 expression is inversely correlated with expression of RH proteins. Together these results suggest that RH1, RH2, and RH3 proteins may work more closely with NH3 to modulate its function.

We observed that NH2 and NH3 RNA expression levels were highly correlated. This should be no surprise because NH2 and NH3 proteins are most homologous to each other forming a clade [24]. NH2 and NH3 functions may differ at the protein level because they behave quite differently in their interactions with the TGA and RH proteins. The same explanation may also apply to explain the fact that NH2 expression levels were also highly correlated with those of RH1, RH2, and RH3.

## Conclusions

We have surveyed the interaction partners of three families of plant innate immunity regulators using Y2H and split YFP assays on a genome-wide scale. Between 70-90% of the time, the Y2H results agree with the split YFP results, indicating a broad and complex interaction network between members of these protein families. Y2H results demonstrate the propensity of these proteins to interact with proteins from other families. The split YFP assay in rice protoplasts shows a higher degree of selectivity in interaction partners for these proteins, while our bridged adaptation of the split YFP assay shows an even greater stringency in interaction partners among the three families of proteins. The interaction profiles of NH, TGA, and RH family members determined by these assays help to explain why NH1 and NH3 play important roles in innate immunity, and also point to a subgroup of TGA proteins (rTGA2.1, rTGA2.2, rTGA2.3, rLG2, TGAL2 and TGAL4) that may be more important to innate immunity than other TGA members. Consistent with their shared role in defense, co-expression analyses reveal that NH1 and NH3 expression patterns show remarkable overlap. NH3 and RH1 are also very tightly coupled in their expression, suggesting an uncharacterized role for RH1 in defense. Unlike NH3, NH1 expression is inversely correlated with expression of RH proteins, suggesting that RH proteins may work more closely with NH3 than with NH1 to modulate its function.

## Methods

### Cloning of cDNAs

Rice cDNA was synthesized from total RNA isolated from Nipponbare rice leaf tissues using the Trizol reagent (Invitrogen). NH1 cDNA was amplified with primers NH1-ATG (5’CACCATGGAGCCGCCGACCA GCCACGTC) and NH1-TAP2 (5’AGCAATGGTGTTCATCTCCTTGGT), NH2 cDNA with primers NH2-ATG (5’CACCATGCCGGCGCGTAGCGCGGTGGT) and NH2-TAP2 (5’CTGTCATTTC TTTGCAACCTTGG), NH3 cDNA with primers NH3-ATG (5’CACCATGGAGACGTCCACCA TAAGCT) and NH3-TAP3 (5’ACTGCAGATTAGACTTAACTGCTG), NH4 cDNA with primers NH4-ATG (5’CACCATGGAGGAAACCCTCAAGTCGCT) and NH4-TAP2 (5’CCACACCCCC TTTCGTCGTCAG), and NH5 cDNA with primers NH5-ATG (5’CACCATGAGCTCCGAGGACT CGCTCA) and NH5-TAP2 (5’TCAACACGGCTAGTAGAAGAGAAG). Individual cDNA was cloned into the pENTR/D vector and sequence confirmed.

NRR cDNA was amplified with primers NRR-ATG (CACCATGGACGCCACCACCA CCGCCAAG) and NRR-TAP2 (TTACTAGTTGTAATCCGTGAGCACCCGCAT), RH1 cDNA with primers RH1-ATG (CACCATGGAGGGAGTTGACGTGAAGGC) and mn133-7 (TTCTCGAGCA AATCAAGACTGGCACATG), RH2 cDNA with primers RH2-ATG (CACCATGGAAGCCCGATTGA GCACGGG) and 133H-2 (TTTACTAGTCTCGAGCCTGATTAATTCATCTGGTCAC), and RH3 cDNA with primers RH3-ATG (CACCATGGATCCCACGATGCCCACTCC) and 133H2-3 (TTTACTAGTCTCGAGACTCATCTGTATGAACTTG). Individual cDNA was cloned into the pENTR/D vector and confirmed by sequence analysis.

rTGA2.1 cDNA was amplified with primers mn1-for (5’CACCGCAGATGCTAGTTCAA GGACTGAC) and mn1-rev (5’CTAGCAAGCCACAGCGAACTCAAA), rTGA2.2 cDNA with primers mn8-for (5’CACCGCAGATGCTAGTTCGAGGACTGAC) and mn8-rev (5’TTACTCCCGT GGCCTAGCAAGCCA), rTGA2.3 cDNA with primers mn38-for (5’CACCCCCTTTGCTGCAGAGT TTGATATG) and mn38-rev (5’CTATTCTTTCGGCCGAGCAAGCCA), rLG2 cDNA with primers mn140-for (5’CACCAGCTCTGTGCGCTACTGCTTGGGC) and mn140-rev (5’T CAAAATCCT GAGTACTGATTCTG). Individual cDNA was cloned into the pENTR/D vector and confirmed by sequence analysis.

TGAL1 cDNA was amplified with primers TGAL1-for (5’CACCATGGAGGGTGGTAGGC TAGGAGGAGCG) and TGAL1-rev (5’TTATTCCCTTGGACGGGCGAGCCA), TGAL2 cDNA with primers TGAL2-for (5’CACCATGGCTGATACAAGTCCAAGGACTGAT) and TGAL2-rev (5’TTATTCCCTTGGACGGGCGAGCCA), TGAL4 with TGAL4-for (5’CACCATGGGAGAAGCTAG CAGTAGTTCAGGA) and TGAL4-rev (5’TCAGAAGGCTGAATATTGGCTCTC), TGAL5 with primers TGAL5-for (5’CACCATGATCCAAAGTGACGCGTACACAGAG) and TGAL5-rev (5’TCAGAAACCGGAGAATTGATTTTG), TGAL6 with primers TGAL6-for (5’CACCATGGGG GCGTACGACCGGCCTCCGCCA) and TGAL6-rev (5’TTAGCTTATCCCTGAATCGCGCGG), TGAL7 with primers TGAL7-for (5’CACCATGGGGGGCTCCAGAGAGGAAGATCGT) and TGAL7-rev (5’CTACATTGCCGGCCCCTCCTCCGG), TGAL8 with primers TGAL8-for (5’CACCATGGCTT ATCCTTCCACCTCTGGCATG) and TGAL8-rev (5’CTAGCCGGCGGCCGGGTGCGGCCG), TGAL9 with primers TGAL9-for (5’CACCATGGCAGAATTGGATCACATCTTCCTC) and TGAL9-rev (5’CTAATTTCTAGGGTTGATGGATGG), and TGAL11 with primers TGAL11-TAP1 (5’CACCGGAGAGGCTAGGAGAGGGCAGAA) and TGAL11-TAP2(5’GAAGGTTAGT CTTCAAAGTCCTTGT). Individual cDNA was cloned into the pENTR/D vector and confirmed by sequence analysis.

### Yeast two-hybrid constructs and assays

Each cDNA was cloned into Gateway-compatible Y2H vectors pLexA and pB42AD by LR recombination (Invitrogen). The Gateway-compatible Y2H vectors pLexA and pB42AD and the Y2H system have been reported previously [21, 45]. Yeast two hybrid constructs were transformed into yeast strain EGY48 and beta-galactosidase activity was assayed by including X-gal in the medium. Beta-galactosidase activity was semi-quantitatively recorded based on the darkness of the blue color. Empty vectors (pLexA and pB42AD) were included as negative controls. For each test, at least three independent yeast colonies were included and results were consistent among the three replicates. Tests were repeated and more colonies were included whenever an outlier occurred.

### Generation of constructs to express non-fusion proteins in protoplasts

cDNA of NH1, NH2, NH3, NH4, and NH5 in pENTR/D vector was cloned into a Gateway-compatible Ubi-pUC vector via LR recombination. Expression of these genes was driven by the maize Ubi-1 promoter.

### Plasmid preparation and protoplast transfection

All plasmid constructs were prepared with a Nucleobond plasmid midi-prep kit from Macherey-Nagel (Bethlehem, PA). For protoplast preparation, 10–14 day old etiolated rice seedlings grown in a sterile ice cream cone were used. Rice protoplast preparation and transfection was done as described before [37, 54].

### Yellow fluorescence protein (YFP) constructs and detection for split YFP assay

Select cDNA was cloned into Gateway-compatible split YFP vectors pY736 (YFPN) and pY735 (YFPC) by LR recombination. The Gateway-compatible split YFP vectors pY736 and pY735 and the split YFP assay have been reported previously [45]. An equal amount of plasmid (5 μg) was used for each protoplast transfection. Empty vectors (pY736 and pY735) were included as negative controls. Rice protoplasts were incubated for 24–36 hr after transfection in incubation buffer. YFP detection used an Axiovert 25 fluorescence microscope (Zeiss) with an excitation wavelength of 500/25 nm and an emission wavelength of 535/30 nm (filter set 46HE). Each split YFP experiment was repeated at least once. The YFP signal strengths were compared between samples and assigned semi-quantitatively according to the results from repeated experiments.

### Bridged split YFP

For each bridged split YFP experiment, a *Ubi-1* promoter-driven, non-YFP fused NH1, NH2, NH3, NH4, or NH5 construct was included, in addition to the YFPN-fused TGA and YFPC-fused RH constructs.

### Detection of protein expressed in rice protoplasts

Rice protoplasts were transfected with YFPN and/or YFPC fusion constructs (8 μg/construct/transfection), together with a Ubi-Gus plasmid (2 μg/transfection) as an internal reference for transfection efficiency. A small aliquot (2 μl out of 240 μl) of the protoplasts was used for GUS activity assay. The remaining transfected rice protoplasts were spun down 24 hours post transfection and re-suspended in 20 μl of 1x SDS protein sample buffer (10% glycerol, 60 mM Tirs-HCl, pH6.8, 2% SDS, 0.01% bromophenol blue, and 1.25% β-mercaptoethanol). The amount of protein loaded in an SDS polyacrylamide gel for each sample was adjusted according to the corresponding GUS activity. YFPN fusion proteins were probed with an α-c-Myc monoclonal antibody (9E11, Santa Cruz Biotechnology) and YFPC fusion proteins probed with an α-HA tag monoclonal antibody (F-7, Santa Cruz Biotechnology).

### Co-expression analysis

Analysis of microarray data for co-expression between two genes was performed according to the method reported before [45].

## Electronic supplementary material

Additional file 1: Figure S1: Yeast two-hybrid pictures for interactions between NH and TGA protein families. Yeast cells containing plasmid constructs expressing proteins as labeled were grown on medium with X-gal for two days. Blue colors indicate an interaction between the two test proteins. The darkness of blue colors is used as the indicator for protein interaction strength. (A) NH proteins were fused to B42AD and TGA proteins fused to LexA. (B) NH proteins were fused to LexA and TGA proteins fused to B42AD. (PPT 1 MB)

Additional file 2: Figure S2: Split YFP pictures for interactions between NH and TGA protein families. Rice protoplast cells were transfected with plasmids expressing proteins as labeled. Fluorescence signals were observed under a fluorescence microscope 20–24 hours after transfection and pictures taken with 2 sec of exposure time. (A) NH proteins were fused to YC and TGA proteins fused to YN. (B) NH proteins were fused to YCNand TGA proteins fused to YC. (PPT 2 MB)

Additional file 3: Figure S3: Yeast two-hybrid pictures for interactions between NH and RH protein families. Yeast cells containing plasmid constructs expressing proteins as labeled were grown on medium with X-gal for two days. Blue colors indicate an interaction between the two test proteins. The darkness of blue colors is used as the indicator for protein interaction strength. (A) NH proteins were fused to LexA and RH proteins fused to B42AD. (B) NH proteins are fused to B42AD and RH proteins fused to LexA. (PPT 374 KB)

Additional file 4: Figure S4: Split YFP pictures for interactions between NH and RH protein families. Rice protoplast cells were transfected with plasmids expressing proteins as labeled. Fluorescence signals were observed under a fluorescence microscope 20–24 hours after transfection and pictures taken with 2 sec of exposure time. (A) NH proteins were fused to YN and RH proteins fused to YC. (B) NH proteins were fused to YC and RH proteins fused to YN. (PPT 912 KB)

Additional file 5: Figure S5: Bridged split YFP pictures for detection of tertiary protein complexes between NH, RH, and TGA families. TGA proteins were fused to YN and RH proteins fused to YC. NH proteins were expressed from the Ubi-1 promoter as a non-fusion protein. Rice protoplast cells were transfected with plasmids expressing proteins as labeled. Fluorescence signals were observed under a fluorescence microscope 20–24 hours after transfection and pictures taken with 2 sec of exposure time. (A) Interaction with YC: NRR. (B) Interaction with YC:RH1. (C) Interaction with YC:RH2. (D) Interaction with YC:RH3. (PPT 3 MB)

Additional file 6: Table S1: Information of Affymetrix microarray experiments. (DOC 68 KB)
