# Supplementary material for: Interaction specificity and coexpression of rice NPR1 homologs 1 and 3 (NH1 and NH3), TGA transcription factors and Negative Regulator of Resistance (NRR) proteins
Source: BMC Genomics. 2014 Jun 11;15(1):461. doi: 10.1186/1471-2164-15-461 (PMC4094623; doi:10.1186/1471-2164-15-461)
Supplement: Supplementary file 1 — Additional file 1: Figure S1: Yeast two-hybrid pictures for interactions between NH and TGA protein families. Yeast cells containing plasmid constructs expressing proteins as labeled were grown on medium with X-gal for two days. Blue colors indicate an interaction between the two test proteins. The darkness of blue colors is used as the indicator for protein interaction strength. (A) NH proteins were fused to B42AD and TGA proteins fused to LexA. (B) NH proteins were fused to LexA and TGA proteins fused to B42AD. (PPT 1 MB) [file 12864_2013_6224_MOESM1_ESM.ppt]

## Slide 1
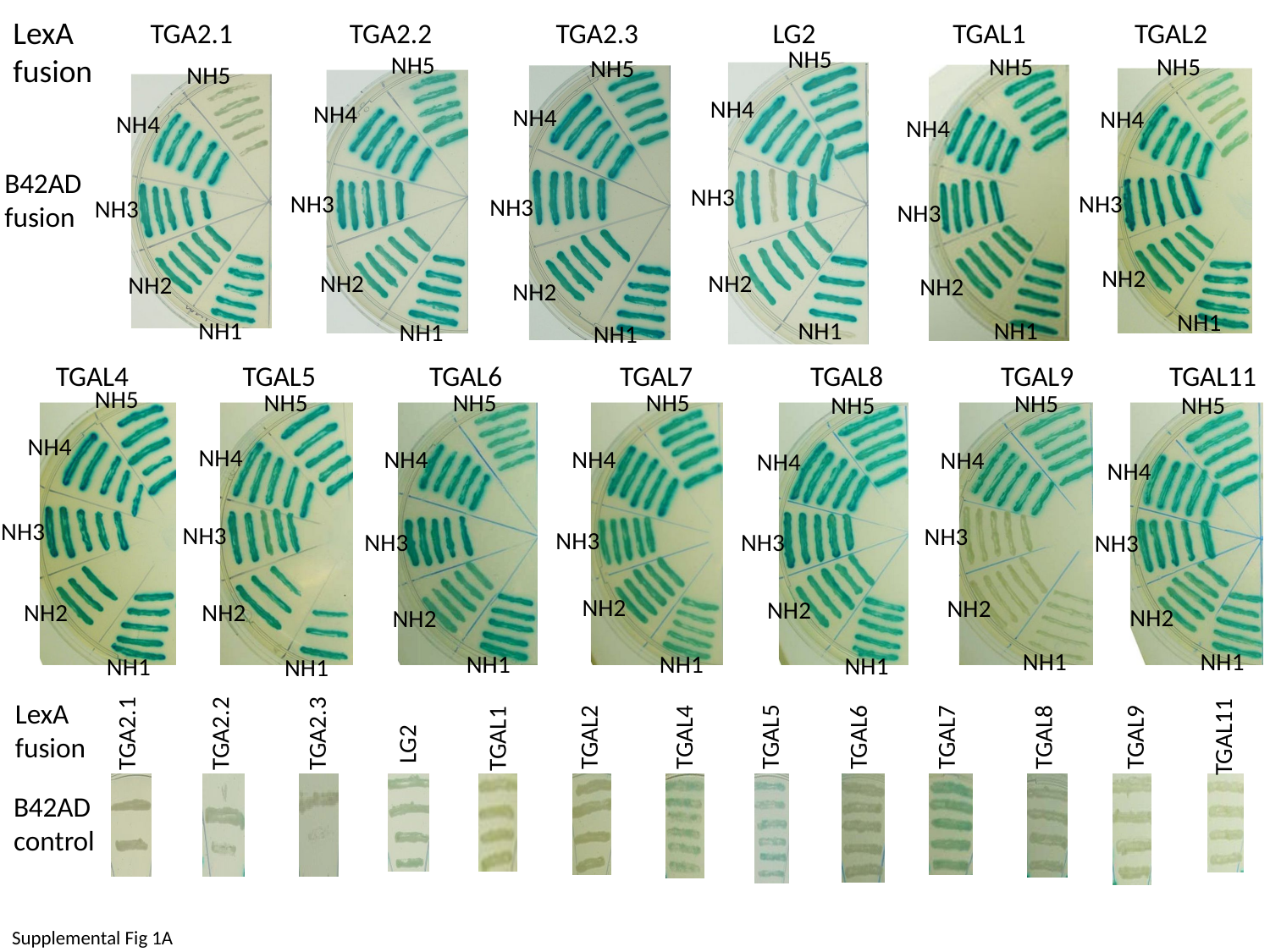

LexA
fusion
TGA2.1
TGA2.2
TGA2.3
LG2
TGAL1
TGAL2
NH5
NH4
NH3
NH2
NH1
NH5
NH4
NH3
NH2
NH1
NH5
NH4
NH3
NH2
NH1
NH5
NH4
NH3
NH2
NH1
NH5
NH4
NH3
NH2
NH1
NH5
NH4
NH3
NH2
NH1
B42AD
fusion
TGAL4
TGAL5
TGAL6
TGAL7
TGAL8
TGAL9
TGAL11
NH5
NH4
NH3
NH2
NH1
NH5
NH4
NH3
NH2
NH1
NH5
NH4
NH3
NH2
NH1
NH5
NH4
NH3
NH2
NH1
NH5
NH4
NH3
NH2
NH1
NH5
NH4
NH3
NH2
NH1
NH5
NH4
NH3
NH2
NH1
LexA
fusion
TGA2.1
TGA2.2
TGA2.3
TGAL5
TGAL2
TGAL4
TGAL6
TGAL7
TGAL8
TGAL9
TGAL11
TGAL1
LG2
B42AD
control
Supplemental Fig 1A

## Slide 2
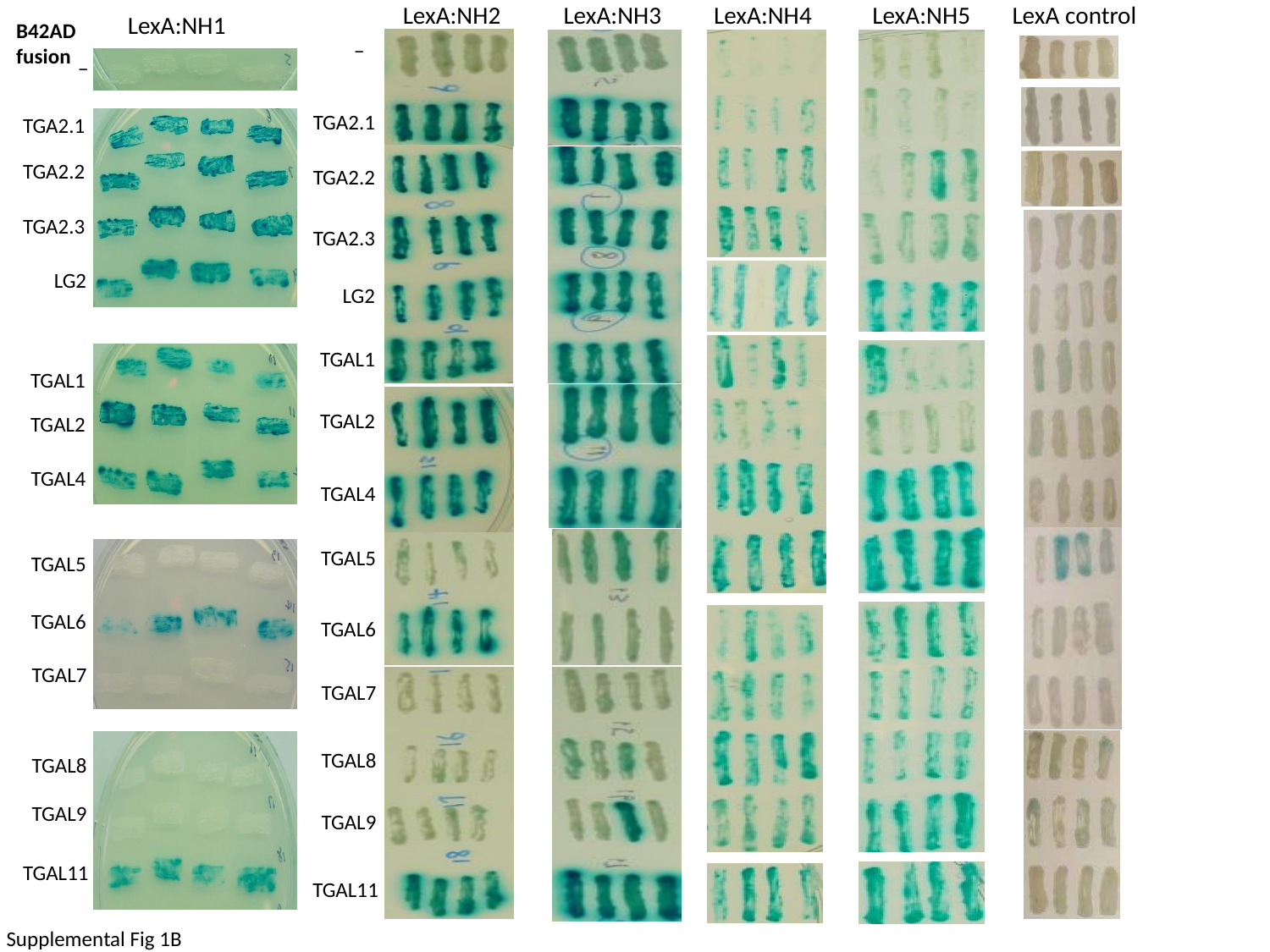

LexA:NH2
LexA:NH3
LexA:NH4
LexA:NH5
LexA control
LexA:NH1
B42AD
fusion
−
−
TGA2.1
TGA2.1
TGA2.2
TGA2.3
LG2
TGA2.2
TGA2.3
LG2
TGAL1
TGAL1
TGAL2
TGAL2
TGAL4
TGAL4
TGAL5
TGAL5
TGAL6
TGAL7
TGAL6
TGAL7
TGAL8
TGAL9
TGAL11
TGAL8
TGAL9
TGAL11
Supplemental Fig 1B
