# Supplementary material for: Interaction specificity and coexpression of rice NPR1 homologs 1 and 3 (NH1 and NH3), TGA transcription factors and Negative Regulator of Resistance (NRR) proteins
Source: BMC Genomics. 2014 Jun 11;15(1):461. doi: 10.1186/1471-2164-15-461 (PMC4094623; doi:10.1186/1471-2164-15-461)
Supplement: Supplementary file 2 — Additional file 2: Figure S2: Split YFP pictures for interactions between NH and TGA protein families. Rice protoplast cells were transfected with plasmids expressing proteins as labeled. Fluorescence signals were observed under a fluorescence microscope 20–24 hours after transfection and pictures taken with 2 sec of exposure time. (A) NH proteins were fused to YC and TGA proteins fused to YN. (B) NH proteins were fused to YCNand TGA proteins fused to YC. (PPT 2 MB) [file 12864_2013_6224_MOESM2_ESM.ppt]

## Slide 1
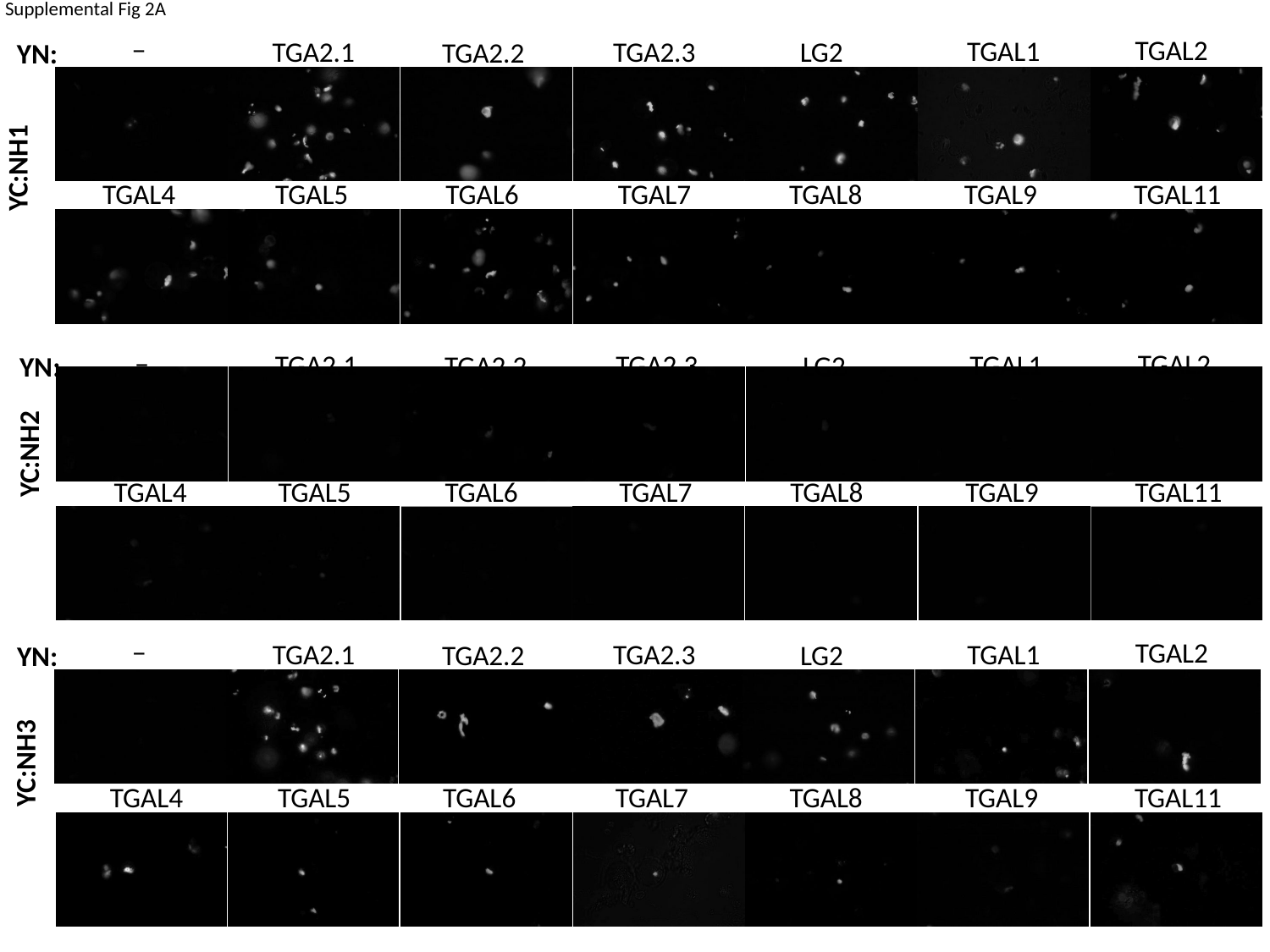

Supplemental Fig 2A
−
TGAL2
TGAL1
TGA2.3
TGA2.1
LG2
TGA2.2
YN:
YC:NH1
TGAL4
TGAL5
TGAL6
TGAL7
TGAL8
TGAL9
TGAL11
−
TGAL2
TGAL1
TGA2.3
TGA2.1
LG2
TGA2.2
YN:
YC:NH2
TGAL4
TGAL5
TGAL6
TGAL7
TGAL8
TGAL9
TGAL11
−
TGAL2
TGAL1
TGA2.3
TGA2.1
LG2
TGA2.2
YN:
YC:NH3
TGAL4
TGAL5
TGAL6
TGAL7
TGAL8
TGAL9
TGAL11

## Slide 2
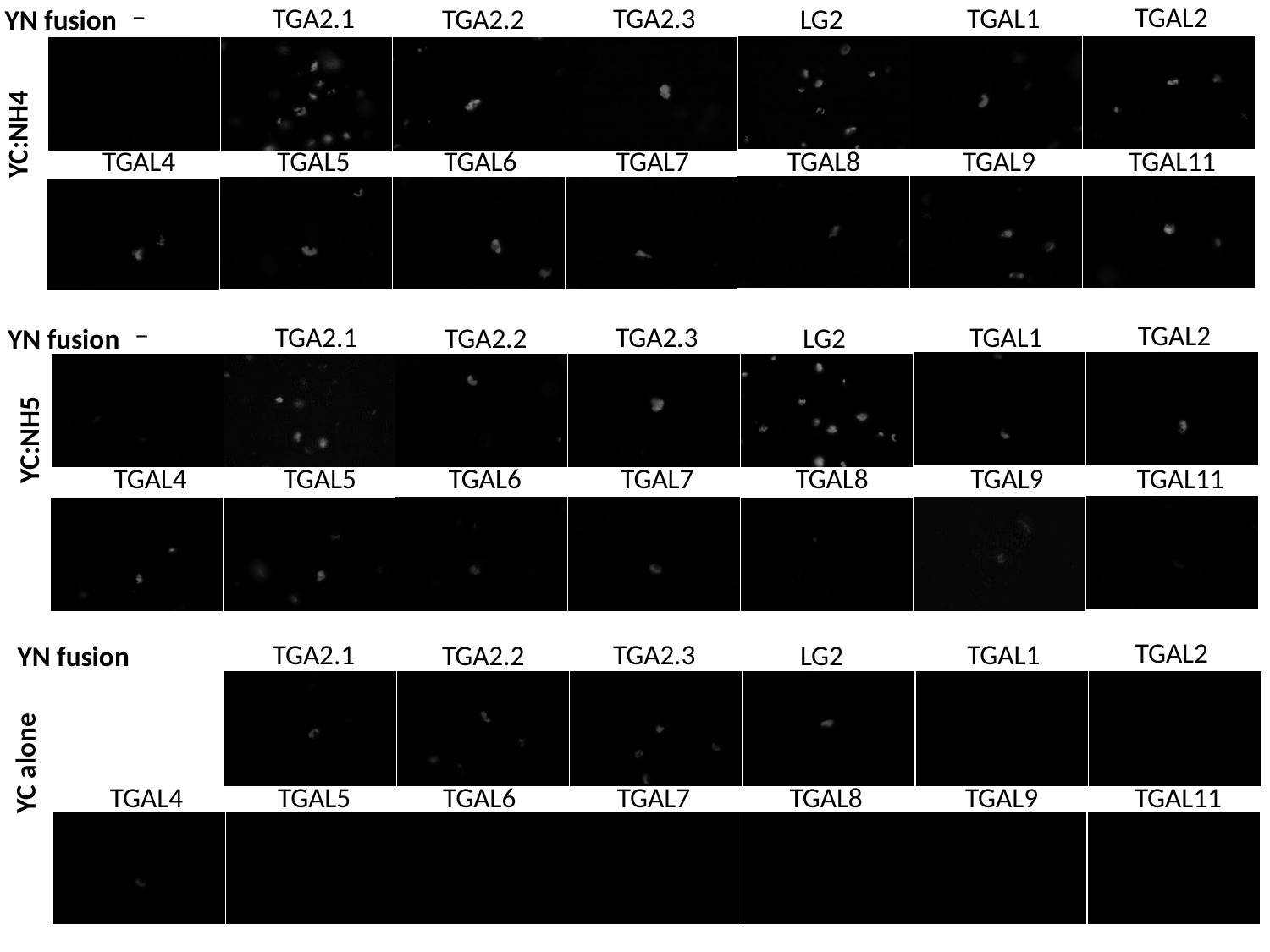

−
TGAL2
TGAL1
TGA2.3
TGA2.1
LG2
TGA2.2
YN fusion
YC:NH4
TGAL4
TGAL5
TGAL6
TGAL7
TGAL8
TGAL9
TGAL11
−
TGAL2
TGAL1
TGA2.3
TGA2.1
LG2
TGA2.2
YN fusion
YC:NH5
TGAL4
TGAL5
TGAL6
TGAL7
TGAL8
TGAL9
TGAL11
TGAL2
TGAL1
TGA2.3
TGA2.1
LG2
TGA2.2
YN fusion
YC alone
TGAL4
TGAL5
TGAL6
TGAL7
TGAL8
TGAL9
TGAL11

## Slide 3
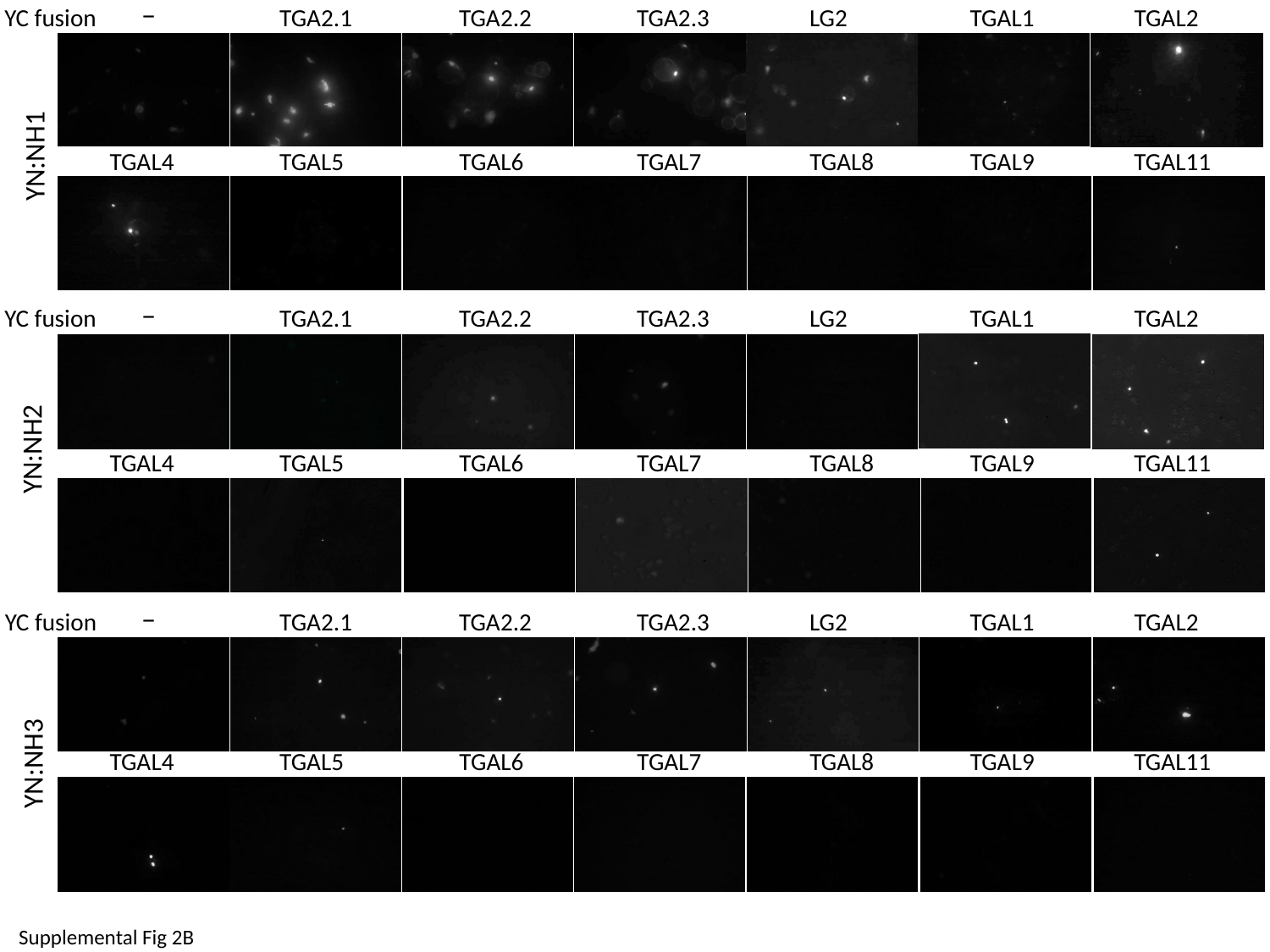

−
YC fusion
TGA2.1
TGA2.2
TGA2.3
LG2
TGAL1
TGAL2
YN:NH1
TGAL4
TGAL5
TGAL6
TGAL7
TGAL8
TGAL9
TGAL11
−
YC fusion
TGA2.1
TGA2.2
TGA2.3
LG2
TGAL1
TGAL2
YN:NH2
TGAL4
TGAL5
TGAL6
TGAL7
TGAL8
TGAL9
TGAL11
−
YC fusion
TGA2.1
TGA2.2
TGA2.3
LG2
TGAL1
TGAL2
TGAL4
TGAL5
TGAL6
TGAL7
TGAL8
TGAL9
TGAL11
YN:NH3
Supplemental Fig 2B

## Slide 4
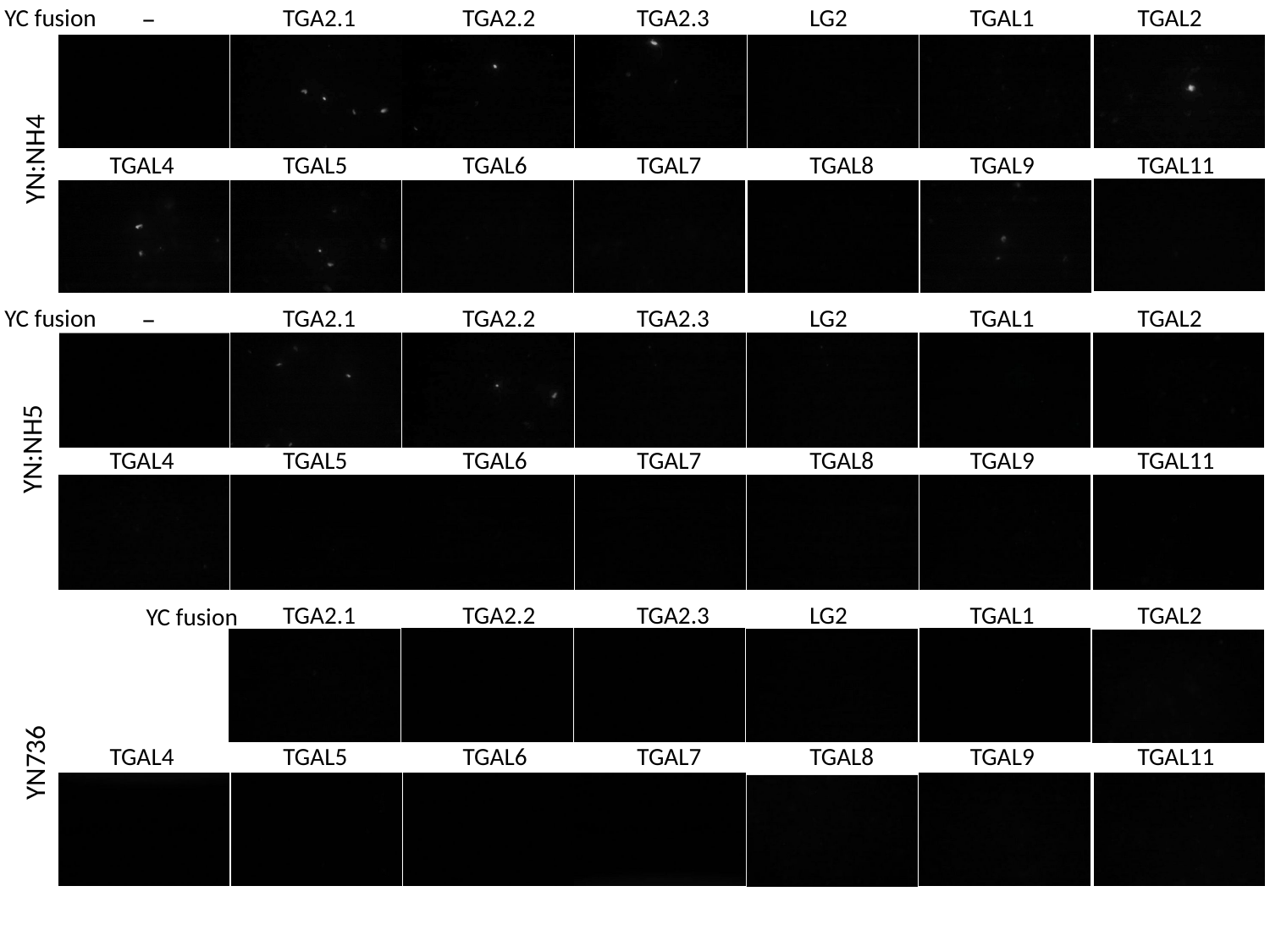

YC fusion
−
TGA2.1
TGA2.2
TGA2.3
LG2
TGAL1
TGAL2
YN:NH4
TGAL4
TGAL5
TGAL6
TGAL7
TGAL8
TGAL9
TGAL11
YC fusion
−
TGA2.1
TGA2.2
TGA2.3
LG2
TGAL1
TGAL2
YN:NH5
TGAL4
TGAL5
TGAL6
TGAL7
TGAL8
TGAL9
TGAL11
TGA2.1
TGA2.2
TGA2.3
LG2
TGAL1
TGAL2
YC fusion
TGAL4
TGAL5
TGAL6
TGAL7
TGAL8
TGAL9
TGAL11
YN736
