# Supplementary material for: Interaction specificity and coexpression of rice NPR1 homologs 1 and 3 (NH1 and NH3), TGA transcription factors and Negative Regulator of Resistance (NRR) proteins
Source: BMC Genomics. 2014 Jun 11;15(1):461. doi: 10.1186/1471-2164-15-461 (PMC4094623; doi:10.1186/1471-2164-15-461)
Supplement: Supplementary file 3 — Additional file 3: Figure S3: Yeast two-hybrid pictures for interactions between NH and RH protein families. Yeast cells containing plasmid constructs expressing proteins as labeled were grown on medium with X-gal for two days. Blue colors indicate an interaction between the two test proteins. The darkness of blue colors is used as the indicator for protein interaction strength. (A) NH proteins were fused to LexA and RH proteins fused to B42AD. (B) NH proteins are fused to B42AD and RH proteins fused to LexA. (PPT 374 KB) [file 12864_2013_6224_MOESM3_ESM.ppt]

## Slide 1
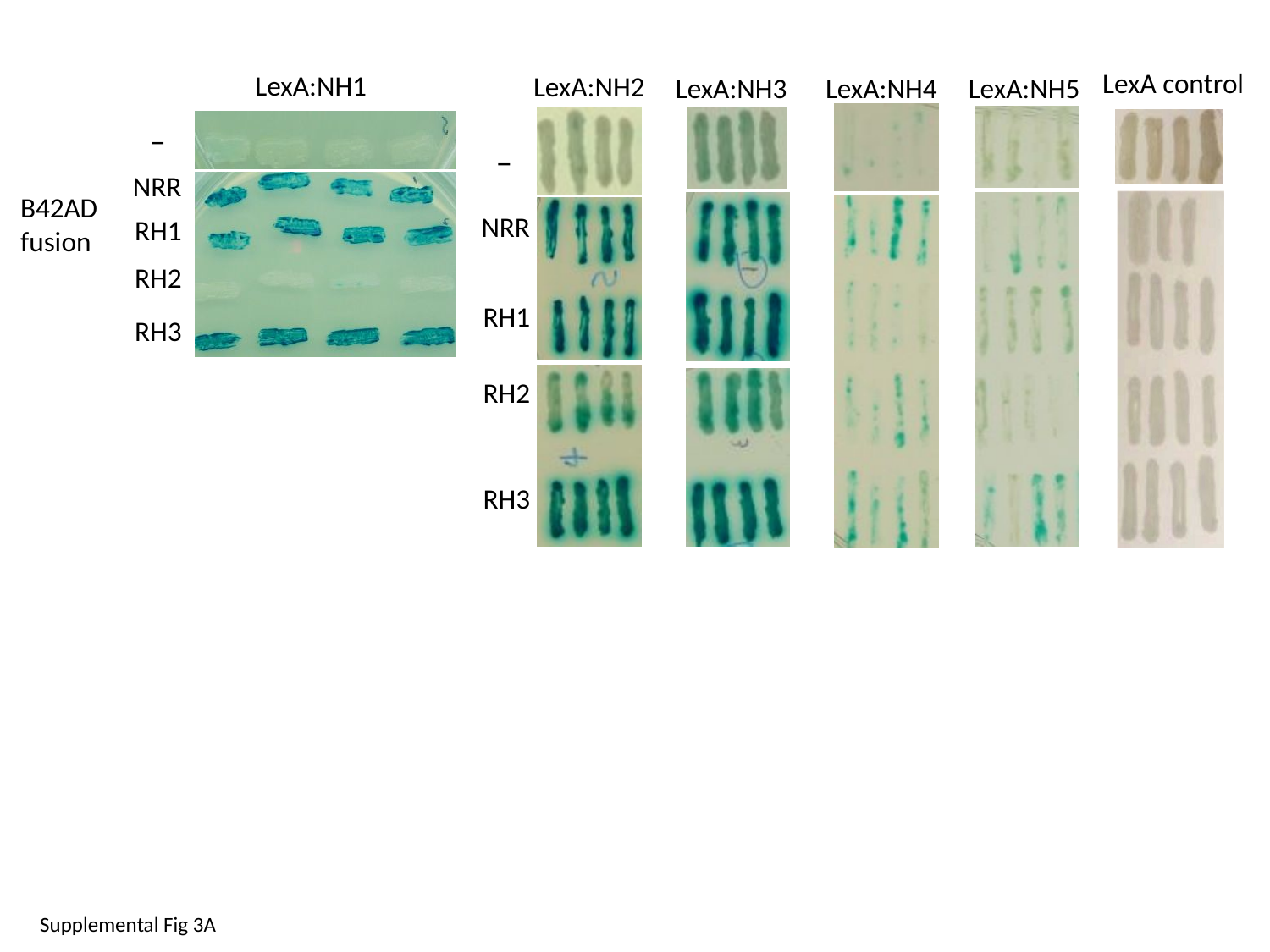

LexA control
LexA:NH1
LexA:NH2
LexA:NH3
LexA:NH4
LexA:NH5
−
−
NRR
B42AD
fusion
NRR
RH1
RH2
RH1
RH3
RH2
RH3
Supplemental Fig 3A

## Slide 2
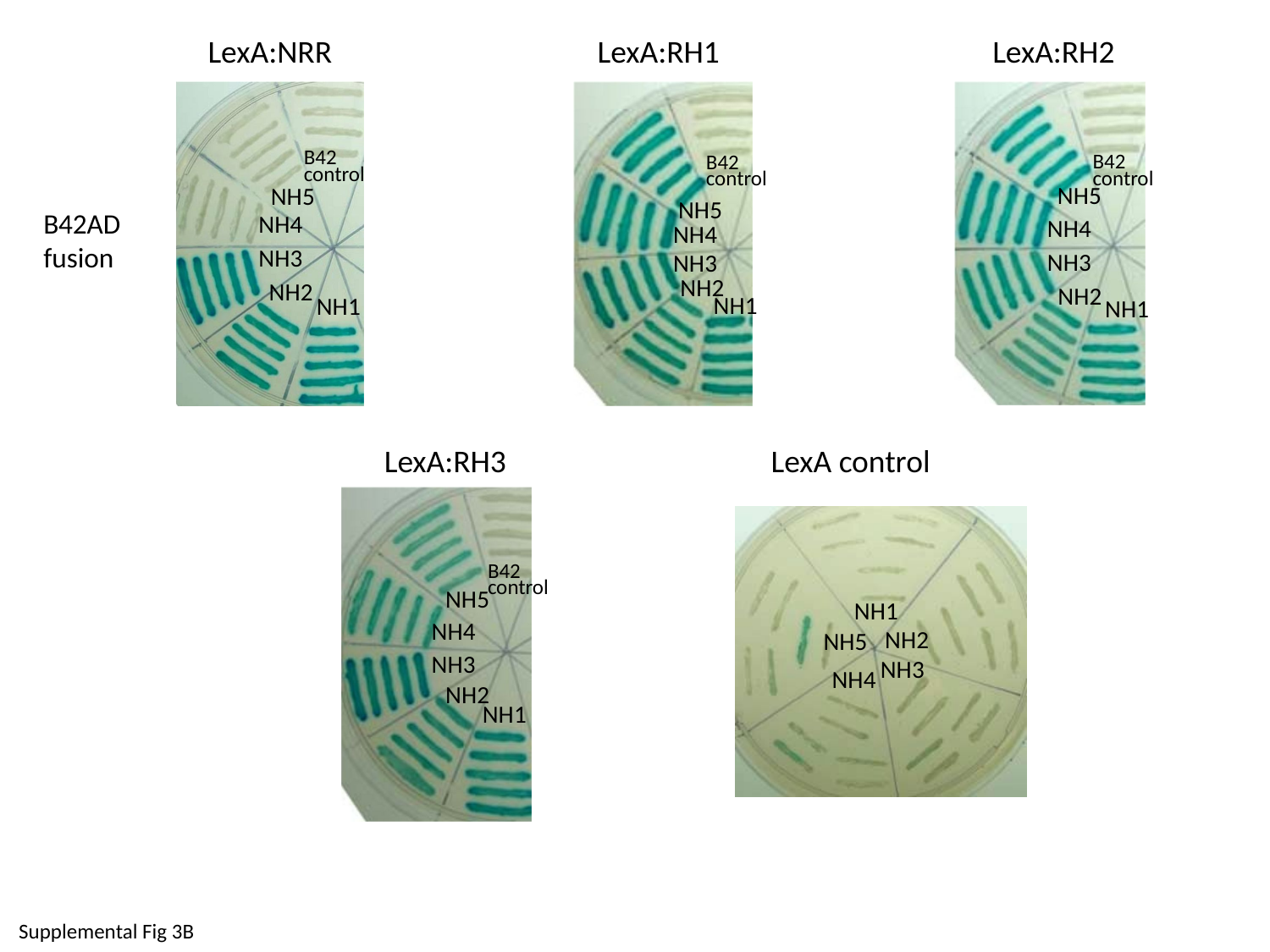

LexA:NRR
LexA:RH1
LexA:RH2
B42
control
B42
control
B42
control
NH5
NH5
NH5
B42AD
fusion
NH4
NH4
NH4
NH3
NH3
NH3
NH2
NH2
NH2
NH1
NH1
NH1
LexA:RH3
LexA control
B42
control
NH5
NH1
NH4
NH2
NH5
NH3
NH3
NH4
NH2
NH1
Supplemental Fig 3B
