# Supplementary material for: Interaction specificity and coexpression of rice NPR1 homologs 1 and 3 (NH1 and NH3), TGA transcription factors and Negative Regulator of Resistance (NRR) proteins
Source: BMC Genomics. 2014 Jun 11;15(1):461. doi: 10.1186/1471-2164-15-461 (PMC4094623; doi:10.1186/1471-2164-15-461)
Supplement: Supplementary file 4 — Additional file 4: Figure S4: Split YFP pictures for interactions between NH and RH protein families. Rice protoplast cells were transfected with plasmids expressing proteins as labeled. Fluorescence signals were observed under a fluorescence microscope 20–24 hours after transfection and pictures taken with 2 sec of exposure time. (A) NH proteins were fused to YN and RH proteins fused to YC. (B) NH proteins were fused to YC and RH proteins fused to YN. (PPT 912 KB) [file 12864_2013_6224_MOESM4_ESM.ppt]

## Slide 1
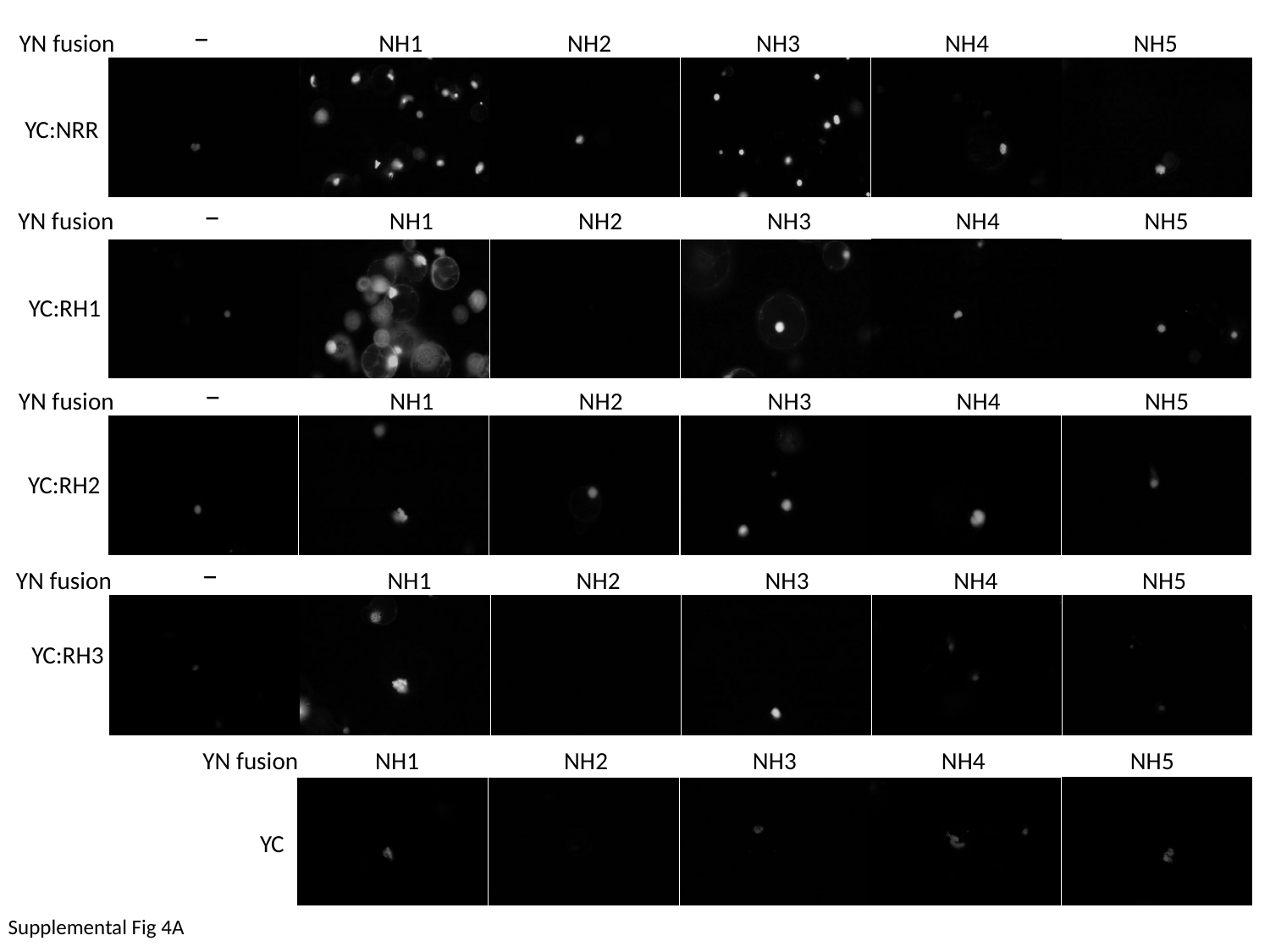

−
YN fusion
NH1
NH2
NH3
NH4
NH5
YC:NRR
−
YN fusion
NH1
NH2
NH3
NH4
NH5
YC:RH1
−
YN fusion
NH1
NH2
NH3
NH4
NH5
YC:RH2
−
YN fusion
NH1
NH2
NH3
NH4
NH5
YC:RH3
YN fusion
NH1
NH2
NH3
NH4
NH5
YC
Supplemental Fig 4A

## Slide 2
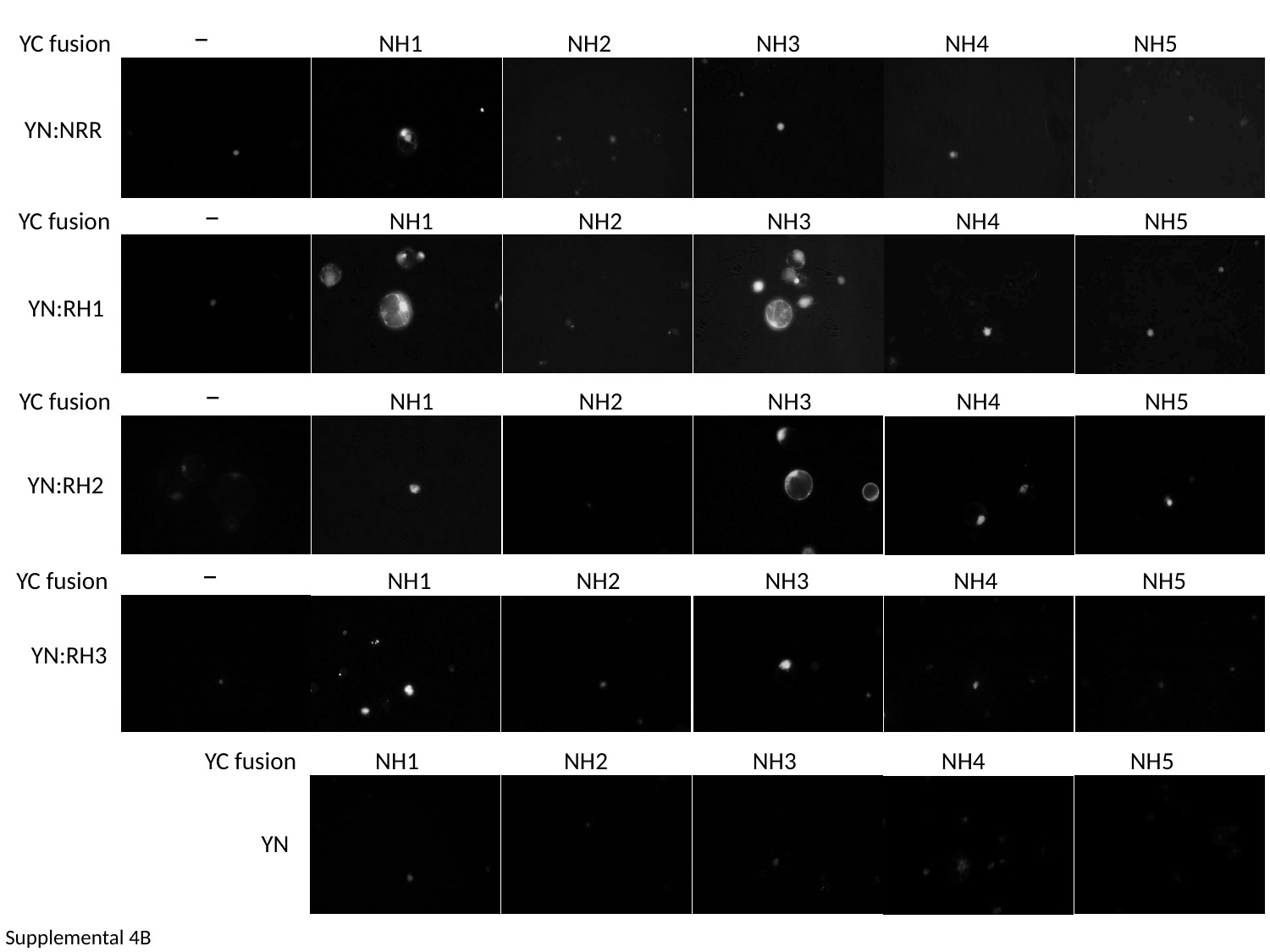

−
YC fusion
NH1
NH2
NH3
NH4
NH5
YN:NRR
−
YC fusion
NH1
NH2
NH3
NH4
NH5
YN:RH1
−
YC fusion
NH1
NH2
NH3
NH4
NH5
YN:RH2
−
YC fusion
NH1
NH2
NH3
NH4
NH5
YN:RH3
YC fusion
NH1
NH2
NH3
NH4
NH5
YN
Supplemental 4B
