# Supplementary material for: Interaction specificity and coexpression of rice NPR1 homologs 1 and 3 (NH1 and NH3), TGA transcription factors and Negative Regulator of Resistance (NRR) proteins
Source: BMC Genomics. 2014 Jun 11;15(1):461. doi: 10.1186/1471-2164-15-461 (PMC4094623; doi:10.1186/1471-2164-15-461)
Supplement: Supplementary file 5 — Additional file 5: Figure S5: Bridged split YFP pictures for detection of tertiary protein complexes between NH, RH, and TGA families. TGA proteins were fused to YN and RH proteins fused to YC. NH proteins were expressed from the Ubi-1 promoter as a non-fusion protein. Rice protoplast cells were transfected with plasmids expressing proteins as labeled. Fluorescence signals were observed under a fluorescence microscope 20–24 hours after transfection and pictures taken with 2 sec of exposure time. (A) Interaction with YC: NRR. (B) Interaction with YC:RH1. (C) Interaction with YC:RH2. (D) Interaction with YC:RH3. (PPT 3 MB) [file 12864_2013_6224_MOESM5_ESM.ppt]

## Slide 1
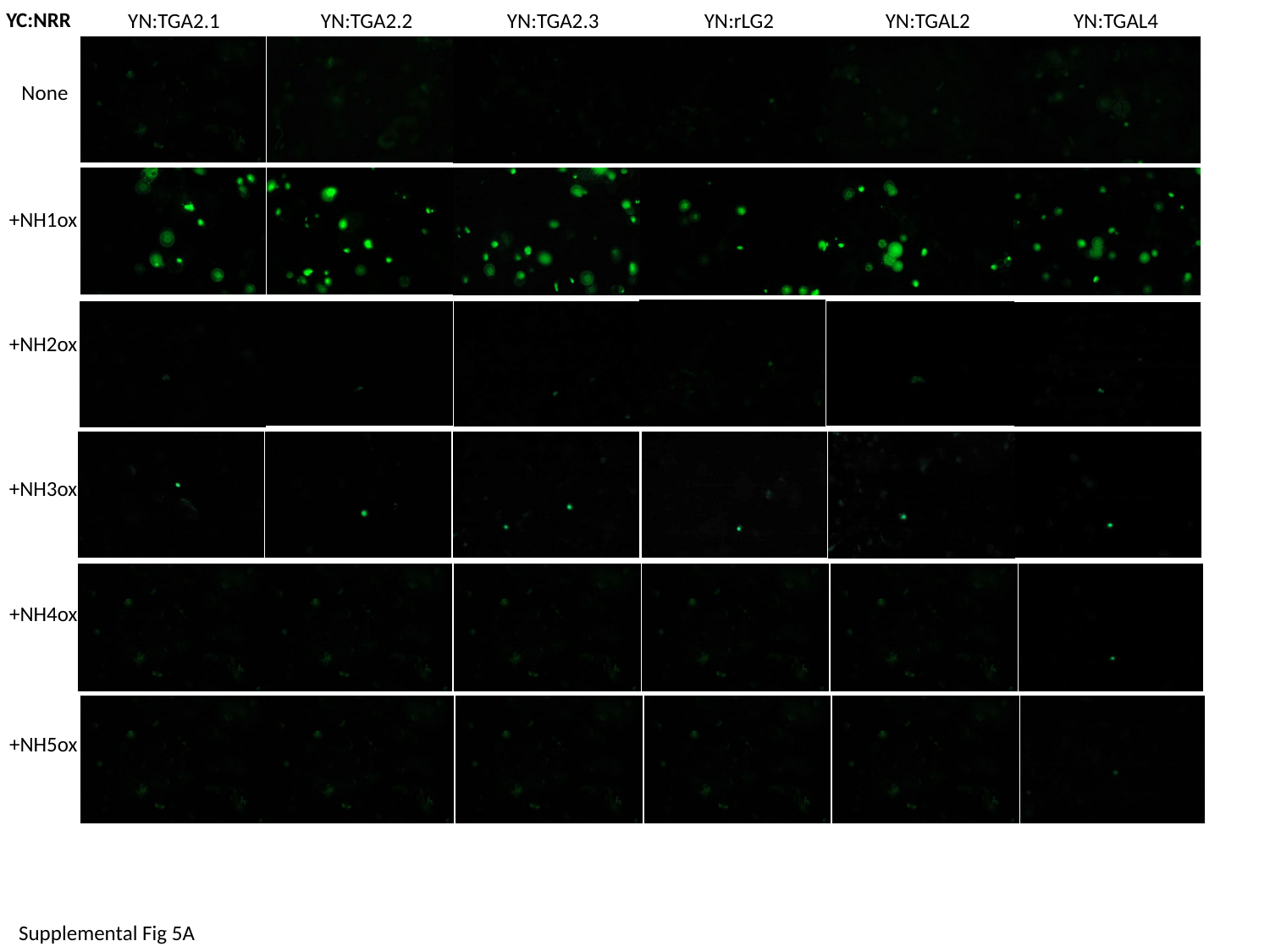

YC:NRR
YN:TGA2.1
YN:TGA2.2
YN:TGA2.3
 YN:rLG2
 YN:TGAL2
 YN:TGAL4
None
+NH1ox
+NH2ox
+NH3ox
+NH4ox
+NH5ox
Supplemental Fig 5A

## Slide 2
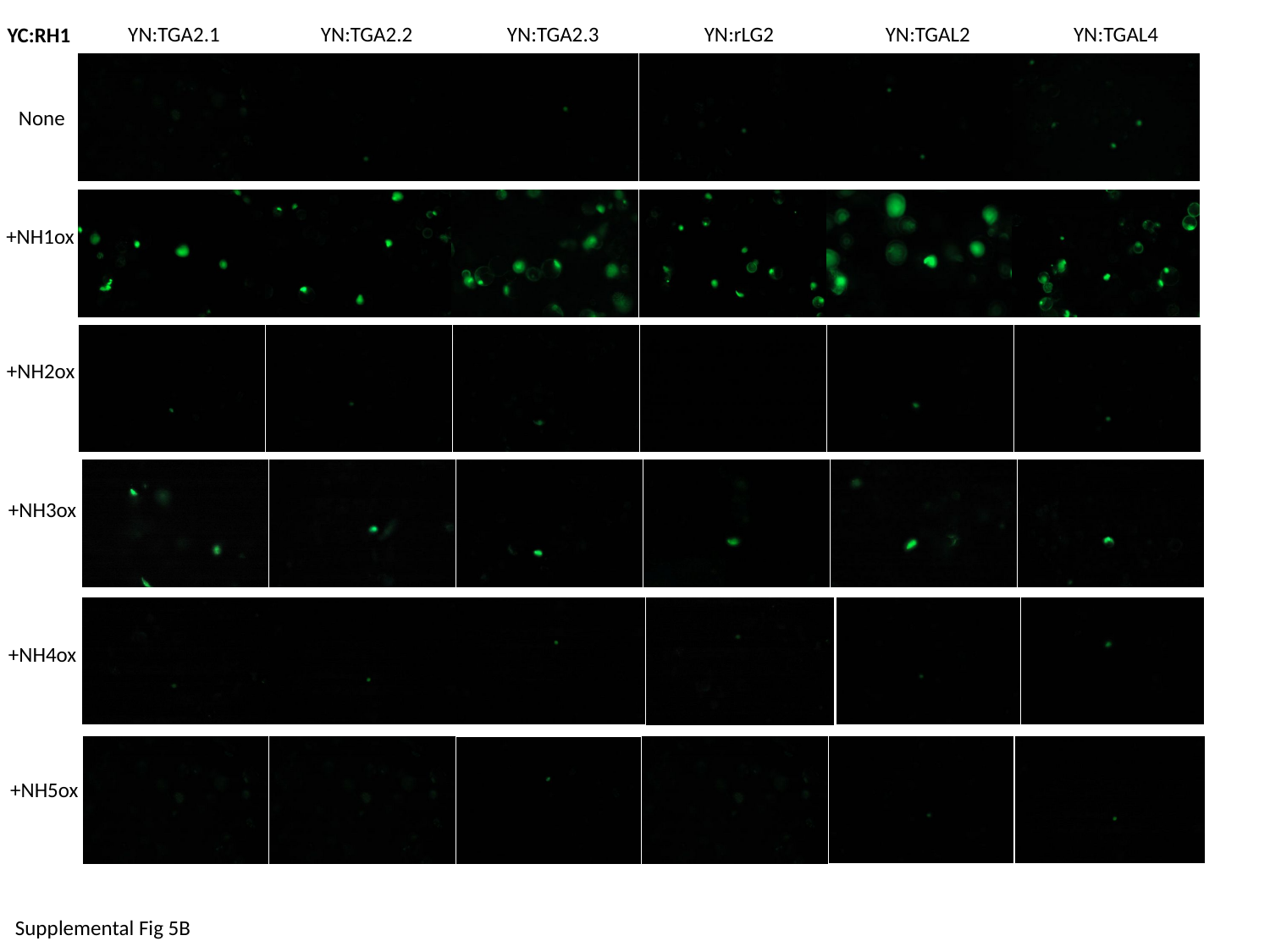

YN:TGA2.1
YN:TGA2.2
YN:TGA2.3
 YN:rLG2
 YN:TGAL2
 YN:TGAL4
YC:RH1
None
+NH1ox
+NH2ox
+NH3ox
+NH4ox
+NH5ox
Supplemental Fig 5B

## Slide 3
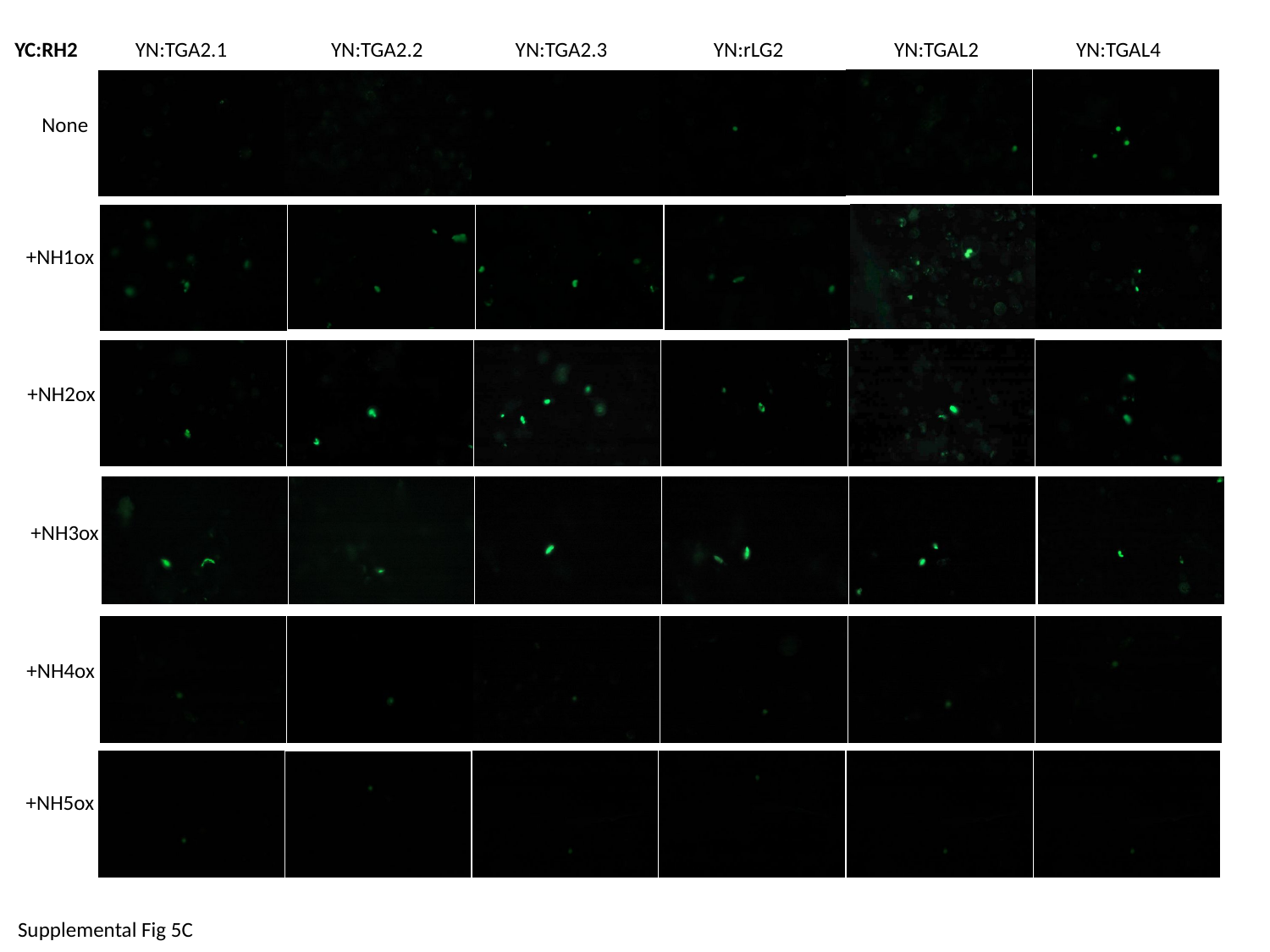

YC:RH2
YN:TGA2.1
YN:TGA2.2
YN:TGA2.3
 YN:rLG2
 YN:TGAL2
 YN:TGAL4
None
+NH1ox
+NH2ox
+NH3ox
+NH4ox
+NH5ox
Supplemental Fig 5C

## Slide 4
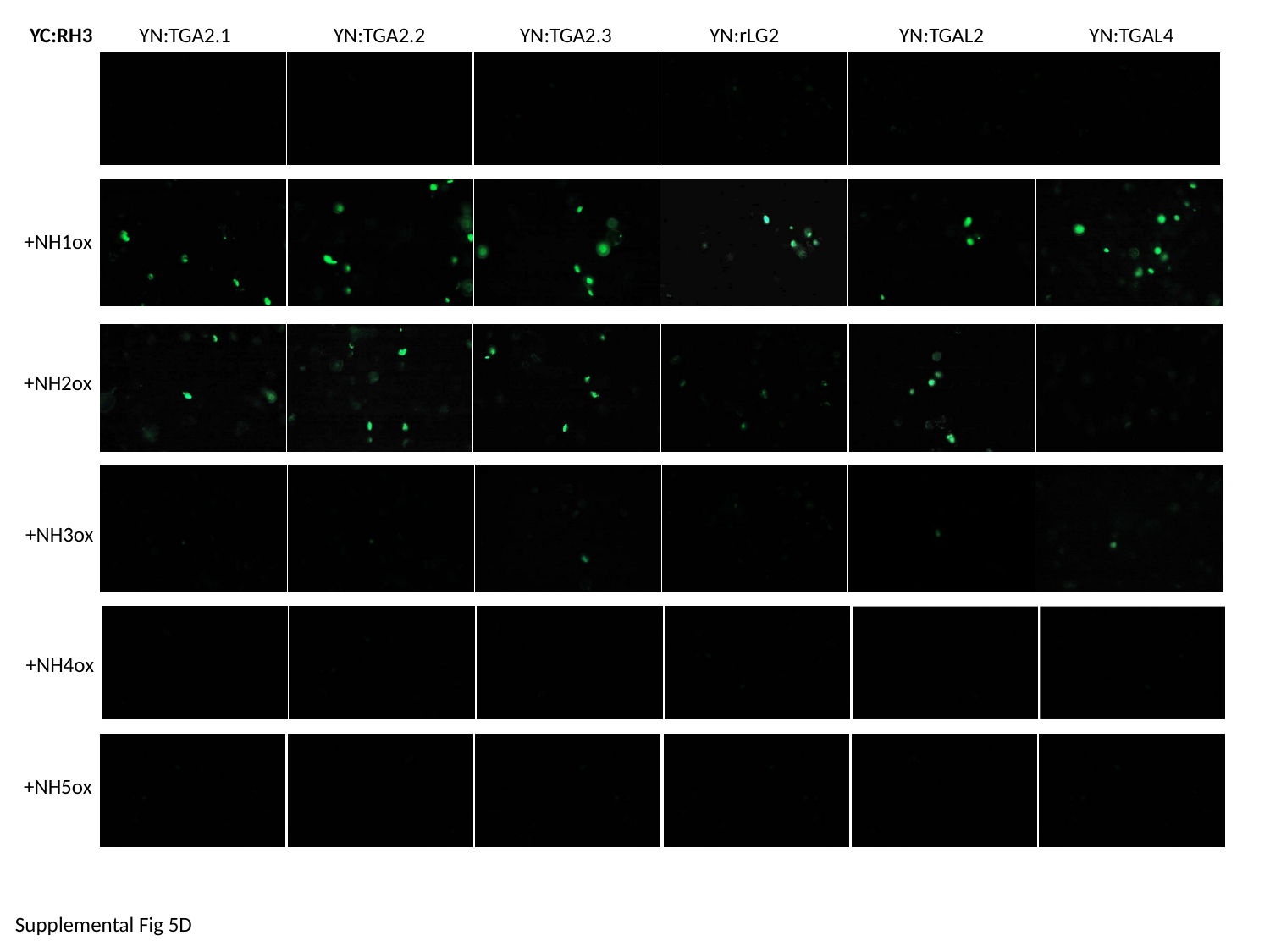

YC:RH3
YN:TGA2.1
YN:TGA2.2
YN:TGA2.3
 YN:rLG2
 YN:TGAL2
 YN:TGAL4
+NH1ox
+NH2ox
+NH3ox
+NH4ox
+NH5ox
Supplemental Fig 5D
