# Supplementary material for: Interaction specificity and coexpression of rice NPR1 homologs 1 and 3 (NH1 and NH3), TGA transcription factors and Negative Regulator of Resistance (NRR) proteins
Source: BMC Genomics. 2014 Jun 11;15(1):461. doi: 10.1186/1471-2164-15-461 (PMC4094623; doi:10.1186/1471-2164-15-461)
Supplement: Supplementary file 6 — Additional file 6: Table S1: Information of Affymetrix microarray experiments. (DOC 68 KB) [file 12864_2013_6224_MOESM6_ESM.doc]

Supplemental Table 1. Information of Affymetrix microarray experiments

| **No.** | **ID** | **Title** | **Contributor** | **Samples** |
| --- | --- | --- | --- | --- |
| 1 | E-MEXP-1766 | The first 24 hours of germination in rice grown under aerobic conditions |  | 15 |
| 2 | E-MEXP-2401 | Nagina-22 (N22) and IR64 under normal and drougth conditions |  | 12 |
| 3 | E-MEXP-2506 | Rice plants grown in different light and temperature cycles |  | 78 |
| 4 | E-MTAB-275 | Rice plants grown in differnent light and temperature conditions |  | 39 |
| 5 | GSE10373 | Rice susceptible and resistant cultivars with the parasitic plant Striga hermonthica | Scholes JD, *et al*. | 24 |
| 6 | GSE10857 | Rice root tips before, at and buckled by a hard layer in two rice varieties | Norton GJ, *et al.* | 12 |
| 7 | GSE11025 | Two contrasting rice genotypes in response to rice stripe virus infection | Zhang X, *et al.* | 12 |
| 8 | GSE12069 | Comparison of transgenic and mutant rice lines | Batista R, *et al.* | 14 |
| 9 | GSE13988 | Rice expression atlas (1): Anther development | Fujita M, *et al.* | 26 |
| 10 | GSE14298 | Rice expression atlas (2): Pollination - Fertilization | Fujita M, et al. | 20 |
| 11 | GSE14299 | Rice expression atlas (3): Early embryogenesis | Fujita M, et al. | 27 |
| 12 | GSE14300 | Rice expression atlas (4): Vegetative tissues | Fujita M, et al. | 25 |
| 13 | GSE14403 | Contrasting rice genotypes in response to salinity stress | Cotsaftis O, *et al.* | 23 |
| 14 | GSE14692 | Temporal regulatory role of miR156 during leaf ontogenesis | Xie K, *et al.* | 12 |
| 15 | GSE15046 | Transcriptome analysis of gibberellin-signaling mutants in rice | Sakakibara H, *et al.* | 12 |
| 16 | GSE15071 | Detection of genomic deletions in rice deletion mutants | Bruce M, *et al.* | 17 |
| 17 | GSE16108 | Parental lines and bulked salt sensitive and salt tolerant RILs | Pandit A, *et al.* | 16 |
| 18 | GSE16341 | Single feature polymorphism detection by Affymetrix expression array | Horiuchi Y, *et al.* | 27 |
| 19 | GSE16793 | Rice undergoing infection by X. oryzae pv. oryzae or by X. oryzae pv. oryzicola | Nino-Liu DO, *et al.* | 60 |
| 20 | GSE17245 | Transcriptome analysis of iron and phosphorus interaction in rice seedlings | Zheng L, *et al.* | 16 |
| 21 | GSE18361 | Rice root infected with Magnaporthe oryzae strain Guy11 | Marcel S, *et al.* | 12 |
| 22 | GSE19024 | Dissecting the developmental transcriptomes of rice | Wang L, *et al.* | 190 |
| 23 | GSE19239 | Transgenic rice line carrying the maize resistance gene Rxo1 to Xoc | Zhou Y, *et al.* | 12 |
| 24 | GSE24048 | Expression data from field droughted rice plants | Price AH, *et al.* | 12 |
| 25 | GSE24228 | Identification of Rhizome-Specific Genes in Oryza longistaminata | Hu F, *et al.* | 15 |
| 26 | GSE25206 | Transcriptomic shifts in rice roots in response to Cr (VI) stress | Dubey S, *et al.* | 14 |
| 27 | GSE26280 | Genome-wide temporal-spatial gene expression profiling of drought responsiveness in rice | Wang D, *et al.* | 36 |
| 28 | GSE4471 | Rice varieties Azucena and Bala grown in 0 and 1ppm arsenate | Norton G, *et al.* | 12 |
| 29 | GSE6737 | Cytokinin treatment and OsRR6-overexpression |  | 28 |
| 30 | GSE6893 | Expression data for reproductive development in rice | Kapoor S, *et al.* | 45 |
| 31 | GSE6901 | Expression data for stress treatment in rice seedlings | Tyagi AK, *et al.* | 12 |
| 32 | OS4 | Rice cultivars IRBB7, IRBB5 and IR24 undergoing infection by X. oryzae pv. oryzae |  | 45 |
